# c-Myb protects cochlear hair cells from cisplatin-induced damage via the PI3K/Akt signaling pathway

**DOI:** 10.1038/s41420-022-00879-9

**Published:** 2022-02-24

**Authors:** Chuan Bu, Lei Xu, Yuechen Han, Man Wang, Xue Wang, Wenwen Liu, Renjie Chai, Haibo Wang

**Affiliations:** 1 Department of Otolaryngology-Head and Neck Surgery, Shandong Provincial ENT Hospital, Cheeloo College of Medicine, Shandong University, Jinan, Shandong China; 2 Shandong Institute of Otorhinolaryngology, Jinan, Shandong China; 3 State Key Laboratory of Bioelectronics, Department of Otolaryngology Head and Neck Surgery, Zhongda Hospital, School of Life Sciences and Technology, Advanced Institute for Life and Health, Jiangsu Province High-Tech Key Laboratory for Bio-Medical Research, Southeast University, Nanjing, 210096 China; 4 Co-Innovation Center of Neuroregeneration, Nantong University, Nantong, 226001 China

**Keywords:** Apoptosis, Mechanisms of disease

## Abstract

The transcription factor c-Myb is vital for cell survival, proliferation, differentiation, and apoptosis. We have previously reported that c-Myb knockdown exacerbates neomycin-induced damage to cochlea cells. However, the function and regulation of c-Myb in the mammalian inner ear remains unclear. Here, we first found that the expression of c-Myb in cochlear HCs was downregulated after cisplatin damage in vivo. Next, to investigate the role of c-Myb in HCs treated with cisplatin, the recombinant virus AAV-ie-CAG-Myb-HA (AAV-c-Myb) that overexpresses c-Myb was constructed and transfected into HCs. The protein expression of c-Myb was effectively up-regulated in cultured cochlear HCs after the virus transfection, which increased cochlear HC viability, decreased HC apoptosis and reduced intracellular reactive oxygen species (ROS) levels after cisplatin injury in vitro. The overexpression of c-Myb in HCs after AAV-c-Myb transfection in vivo also promoted HC survival, improved the hearing function of mice and reduced HC apoptosis after cisplatin injury. Furthermore, c-Myb-HC conditional knockout mice (Prestin; c-Myb-cKO) in which c-Myb expression is downregulated only in cochlear OHCs were generated and the cisplatin-induced HCs loss, apoptosis and hearing deficit were all exacerbated in Prestin; c-Myb-cKO mice treated with cisplatin in vivo. Finally, mechanistic studies showed that upregulation of the PI3K/Akt signaling pathway by c-Myb contributed to the increased HC survival after cisplatin exposure in vitro. The findings from this work suggest that c-Myb might serve as a new target for the prevention of cisplatin-induced HC damage and hearing loss.

## Introduction

Hearing loss is a common disabling disease affecting up to 466 million people worldwide, of which sensorineural hearing loss (SNHL) accounts for the vast majority of cases [1]. Cisplatin is a platinum-containing anticancer drug that is widely used clinically to treat various human malignant tumors [2], but the clinical use of cisplatin is limited by its toxicity, including nephrotoxicity, ototoxicity, etc. [3, 4]. Cisplatin can cause serious damage to cochlear hair cells (HCs), spiral ganglion neurons (SGNs), and the stria vascularis [5, 6] and thus can cause bilateral, progressive, and irreversible SNHL [7]. Although the ototoxicity induced by cisplatin has been attributed to the formation of double-stranded breaks in DNA, the accumulation of reactive oxygen species (ROS), mitochondrial damage, apoptosis [7–11], and the stimulation of inflammatory responses [12, 13], the exact mechanism of cisplatin ototoxicity is not fully understood, and there are currently no methods for protecting against ototoxicity during cisplatin-based chemotherapy.

Myb genes are a family of transcription factors that are implicated in the control of the proliferation and differentiation of both normal and transformed cells, among which c-Myb is the best-characterized member. c-Myb is vital for survival, and knockout of both alleles of the gene results in lethality at embryo day 14 in mice [14, 15], c-Myb has been shown to modulate cell proliferation, differentiation, and apoptosis via protein-protein interactions and through transcriptional regulation of signaling pathways, including phosphatidylinositol 3 kinase (PI3K)/Akt, NF-κB, Wnt, etc. [16–19]. In research in the inner ear, in situ hybridization has shown that c-Myb is expressed within the chicken otic placode and it might play a role in otic development [20]. Recently, we found that c-Myb is dynamically expressed in mouse cochlea HCs and showed that disruption of c-Myb resulted in the induction of apoptosis, the accumulation of reactive oxygen species (ROS), and the reduction of Bcl-2 expression in HEI-OC1 cells, a HC-like cell line, in response to neomycin injury [21]. However, the role of c-Myb in cisplatin-induced ototoxicity, as well as the underlying molecular mechanisms, remain unclear.

Thus, the aim of this study was to determine whether c-Myb is protective against cisplatin-induced ototoxicity in cultured HCs in vitro and in adult mouse cochlear HCs and, if so, the possible mechanisms underlying this action.

## Results

### The expression of c-Myb in cochlear HCs is downregulated after cisplatin exposure in vivo

**Fig. 1:**
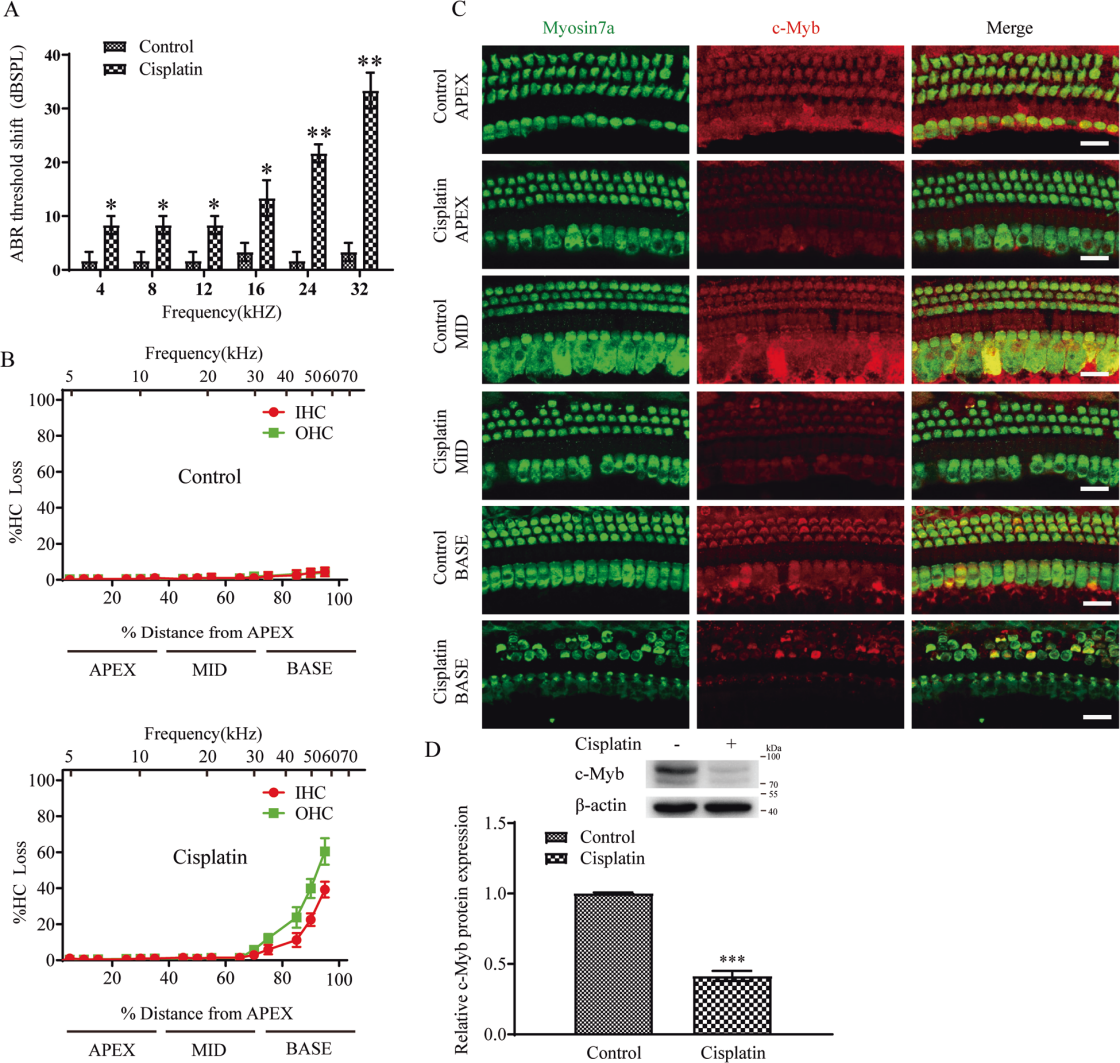
The expression of c-Myb in cochlear HCs is downregulated after cisplatin exposure. **A** The ABR thresholds shift of all frequencies were increased after cisplatin administration in mice compared to the control group. **B** Cell counting and cochleogram there was a significant HCs loss in the cochlear basal turn of the cisplatin group compared to that of the control group, but no significant HCs loss was found in the apical and middle turns in the cisplatin group versus control group. **C** Representative immunostaining images showing that the fluorescence intensity of c-Myb was significantly downregulated after cisplatin administration compared with the control group. Scale bar = 20 μm. **D** Western blot showed that the protein level of c-Myb was significantly downregulated in the cisplatin group compared with the control group. *n* = 6 for each group. *p < 0.05, **p < 0.01, and ***p < 0.001.

Our previous study showed that the expression of c-Myb gradually increases with age in mouse cochlear HCs [21], and strong expression of c-Myb is observed in outer HCs (OHCs) at postnatal day (P) 14, while only weak expression is seen in the inner HCs (IHCs) at this time, and c-Myb is strongly expressed in both IHCs and OHCs starting from P30. The cisplatin administration in vivo was performed according to our previous report [11], and the auditory brainstem response (ABR) thresholds shift at all frequencies were increased after cisplatin administration in mice compared to the control group (Fig. 1A). Immunostaining and cell counting showed that there was a significant HCs loss in the cochlear basal turn of the cisplatin group compared to that of the control group, as there was 16.372 ± 6.620 % IHCs and 28.406 ± 9.882 % OHCs missing compared with the controls (Fig. 1B, C, Supplementary Table 1), but no significant HCs loss was found in the apical and middle turns in the cisplatin group versus control group. Immunostaining showed that after cisplatin exposure the fluorescence intensity of c-Myb in the cisplatin group was significantly reduced compared to that of the control group (Fig. 1C). Western blot also showed that the protein level of c-Myb was significantly decreased in the cisplatin group compared to the control group (Fig. 1D). Together, these results suggest that the expression of c-Myb in cochlear HCs is significantly downregulated after cisplatin exposure in adult mice, thus indicating that c-Myb might play a role in cisplatin-induced HC damage.

### c-Myb overexpression increases cochlear HC viability after cisplatin damage in vitro

The recombinant virus AAV-mNeonGreen-c-Myb-HA (AAV-c-Myb) that overexpresses c-Myb was constructed using the AAV vector AAV-ie-CAG-mNeonGreen (AAV-ie), which is reported to efficiently infect HCs [22]. The efficiency of virus transfection in HCs in vitro was determined firstly. As indicated by the quantification of mNeonGreen reporter, there was 83.250 ± 0.944%, 80.470 ± 2.976%, and 76.600 ± 2.804% of the HCs in the apical, middle, and basal turns were positively transfected, respectively (Supplementary Fig. 1A, B). The number counting of HCs showed that there was no significant difference in HC number between the AAV-ie group and the control group (Supplementary Fig. 1C, D). Western blot showed that the level of c-Myb protein in the AAV-c-Myb transfection group was significantly increased compared to the control group (Supplementary Fig. 1E), indicating that the expression of c-Myb can be effectively up-regulated in cultured cochlear HCs.

**Fig. 2:**
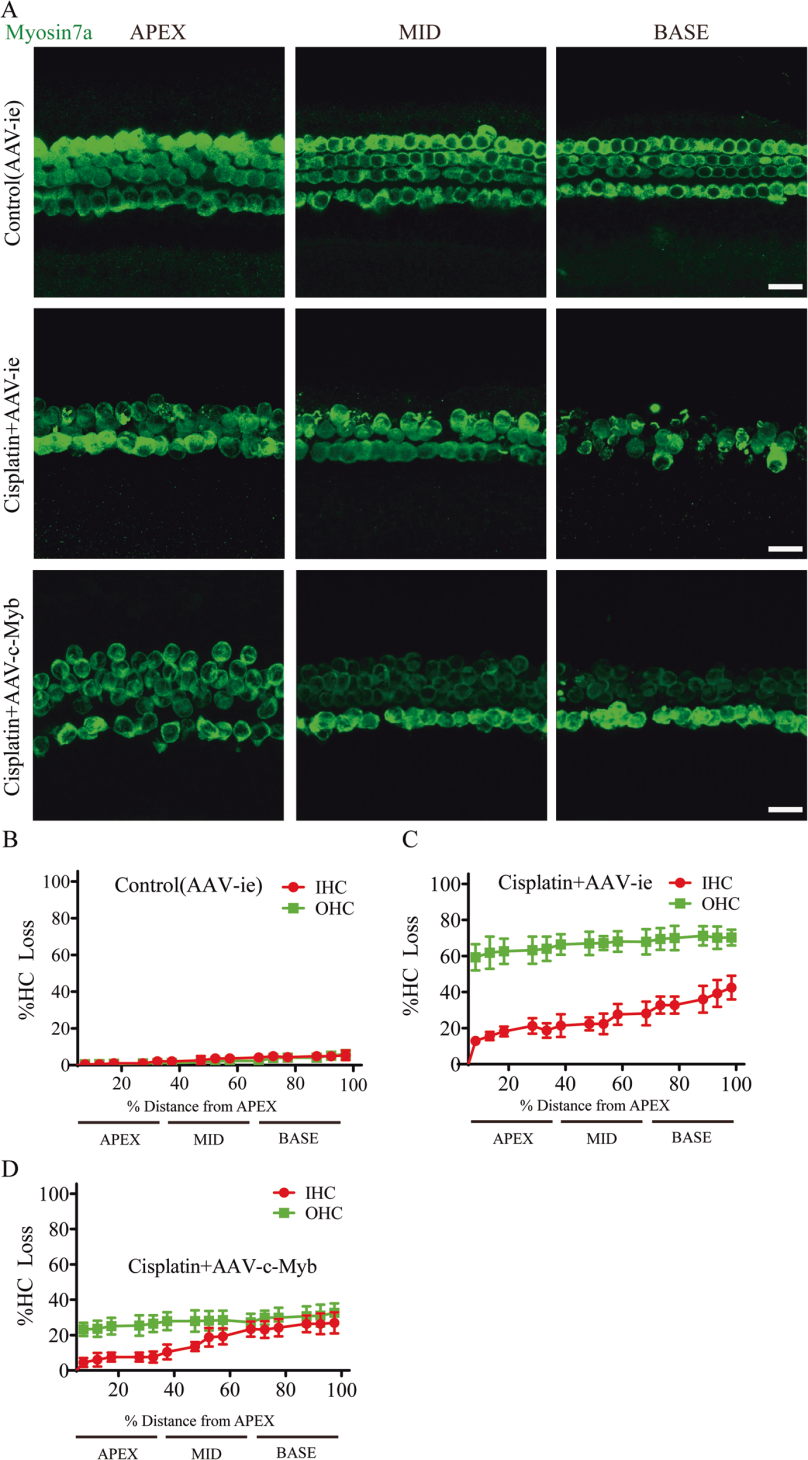
Overexpression of c-Myb increases cochlear HC viability after cisplatin exposure. **A**–**D** Immunofluorescence staining and cell counting showed that cisplatin and AAV-ie co-treatment caused significant IHCs and OHCs loss in all three turns compared to control (AAV-ie) group. The loss of IHCs and OHCs in all three turns were less prominent in cisplatin + AAV-c-Myb group compared with the cisplatin + AAV-ie group. Scale bar = 20 μm.***p < 0.001, *n* = 6 for each group.

We next studied the effect of c-Myb on cochlear HC viability after cisplatin exposure in vitro. Cultured cochleae were pre-treated with either 2 × 10^10^ GCs empty vector AAV-ie or the virus of AAV-c-Myb for 12 h and then continued to be co-treated with or without 30 μM cisplatin [23] for 48 h. Immunostaining and cell counting showed that cisplatin and AAV-ie co-treatment caused significant IHCs and OHCs loss in all three turns compared to control (AAV-ie) group (Fig. 2A–C, Supplementary Table 2), and the loss of OHCs is more serious than that of IHCs, which is consistent with previous studies showing that cisplatin preferentially damages OHCs [6, 24, 25]. Meanwhile, the loss of IHCs and OHCs in all three turns were less prominent in cisplatin + AAV-c-Myb group compared with the cisplatin + AAV-ie group (Fig. 2C and D, Supplementary Table 2), suggesting that the overexpression of c-Myb in cochlear HCs significantly increased cell viability of both IHCs and OHCs after cisplatin exposure in vitro.

### c-Myb upregulation protects cochlear HCs against apoptosis in vitro

**Fig. 3:**
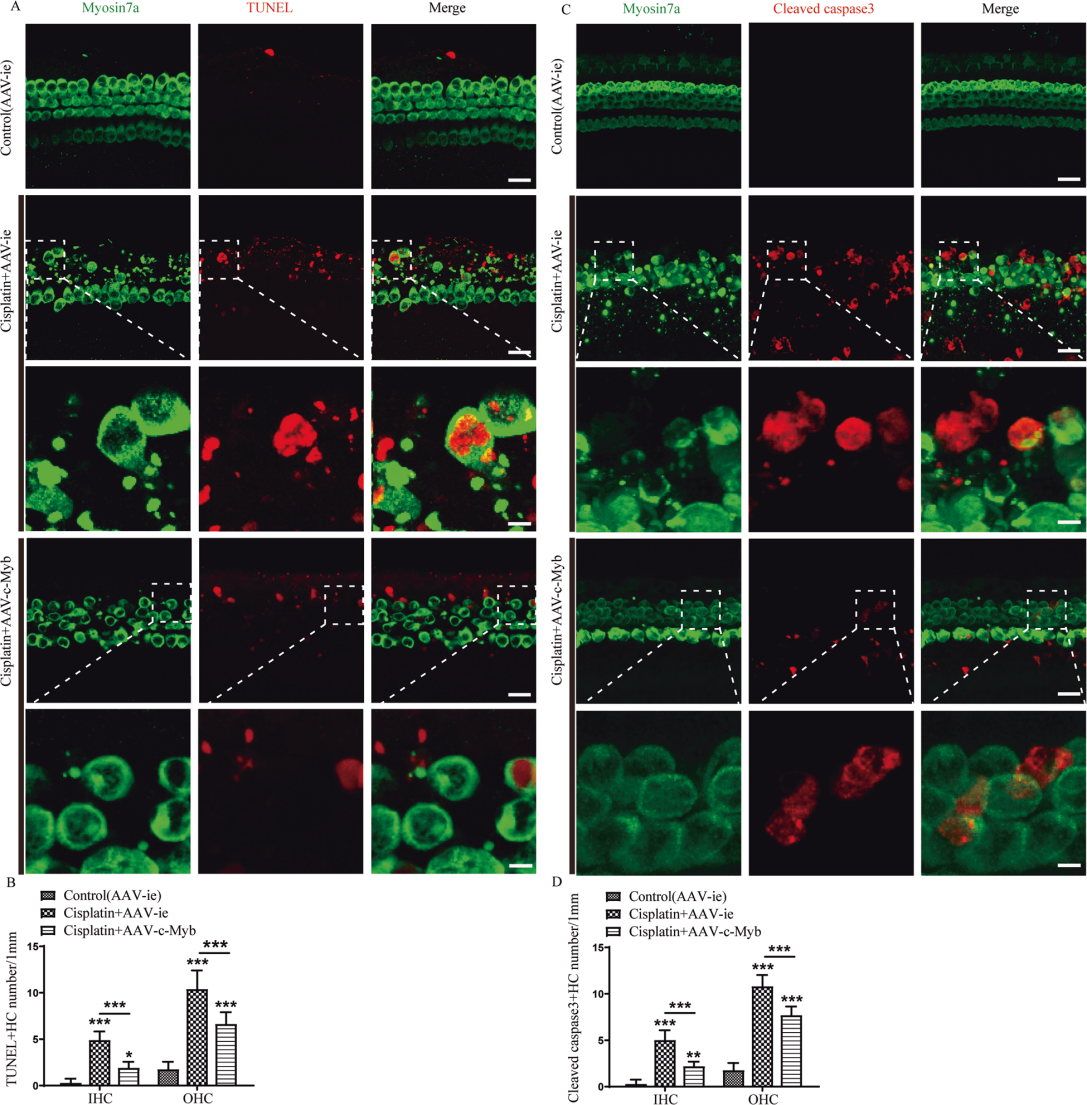
c-Myb overexpression decreases cochlear HC apoptosis induced by cisplatin. **A** Representative images of TUNEL staining showing that apoptotic cochlear HCs exhibited both TUNEL (red) and myosin7a (green) fluorescence after cisplatin treatment. Scale bar (low magnification) = 20 μm. Scale bar (high magnification) = 5 μm. **B** Statistical analysis verified that the numbers of TUNEL/myosin 7a double-positive IHCs and OHCs in the cisplatin + AAV-c-Myb group were reduced significantly compared to the cisplatin + AAV-ie group. **C** Representative images of cleaved-caspase3 staining showing that cleaved-caspase3 fluorescence (red) was present in the apoptotic HCs (myosin 7a labeling, green) after cisplatin exposure. Scale bar (low magnification) = 20 μm. Scale bar (high magnification) = 5 μm. **D** Cell counting and statistical analysis confirmed that the number of cleaved-caspase3/myosin 7a double-positive IHCs and OHCs in the cochlear basal turns was increased significantly after cisplatin treatment compared to the vehicle control group, whereas the number of double-positive cells was decreased significantly in the cisplatin + AAV-c-Myb group compared with the cisplatin + AAV-ie group. **p < 0.01, ***p < 0.001. *n* = 6 for each group.

The Terminal deoxynucleotidyl transferase-mediated dUTP nick-end-labeling (TUNEL) assay and cleaved-caspase 3 immunostaining were used to detect apoptosis in cultured HCs. According to a previous publication [26] and our results in Fig. 1B, which showed that the HCs in the basal turn are the most vulnerable to cisplatin injury, we therefore chosen the basal turn cochlear HCs to analyze in the subsequent in vitro experiments. As shown in Fig. 3A, apoptotic cochlear HCs exhibited both clear TUNEL (red) and myosin 7a (green) fluorescence after treatment with cisplatin, and the numbers of TUNEL/myosin 7a double-positive IHCs and OHCs in the cisplatin + AAV-c-Myb group were reduced significantly compared to the cisplatin + AAV-ie group (Fig. 3B, Supplementary Table 3). Immunostaining showed cleaved-caspase3 fluorescence (red) in the apoptotic HCs (myosin 7a labeling, green) after cisplatin exposure (Fig. 3C), the numbers of Cleaved-caspase3/myosin 7a double-positive IHCs and OHCs were decreased significantly in the cisplatin + AAV-c-Myb group compared with the cisplatin + AAV-ie group (Fig. 3D, Supplementary Table 3). These results suggest that exposure to cisplatin induced apoptosis in HCs and that the overexpression of c-Myb prevented HC apoptosis in vitro.

### Upregulating c-Myb expression decreases intracellular ROS levels after cisplatin exposure in vitro

**Fig. 4:**
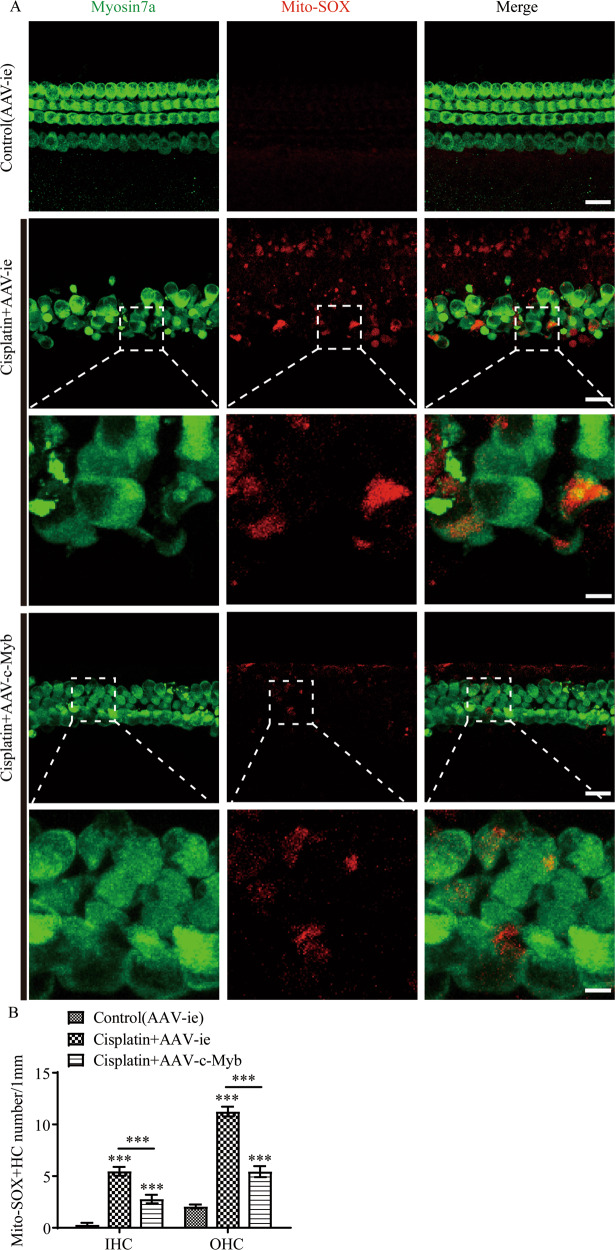
The upregulation of c-Myb expression reduces the level of ROS in cochlear HCs after cisplatin exposure. **A** Representative images of Mito-SOX Red staining showing the accumulation of ROS as indicated by the intensified immunostaining signals of Mito-SOX Red (red) in cultured HCs (myosin 7a, green). Scale bar (low magnification) = 20 μm. Scale bar (high magnification) = 5 μm. **B** A significant number of Mito-SOX/myosin 7a double-positive IHCs and OHCs were observed after cisplatin treatment for 48 h in vitro, while pretreatment with AAV-c-Myb significantly reduced the number of cisplatin-induced Mito-SOX Red/myosin 7a double-positive HCs in the cultured basal-turn cochlear HCs. ***p < 0.001, *n* = 6 for each group.

The accumulation of ROS was detected by the intensified immunostaining signals of Mito-SOX Red (red) in cultured HCs (myosin 7a labelling, green). A significant number of Mito-SOX/myosin 7a double-positive IHCs and OHCs were observed after cisplatin treatment, and pretreatment with AAV-c-Myb significantly reduced the number of the double-positive HCs (Fig. 4A and B, Supplementary Table 3), suggesting that upregulation of c-Myb significantly reduced intracellular ROS levels in HCs after cisplatin injury.

### Overexpression of c-Myb in cochlear HCs in vivo improves HC survival and hearing function, protects ribbon synapse in mice after cisplatin injury

The mice were injected with AAV-ie to detect the virus transfection efficiency in HCs in vivo. The ABR results showed no significant changes in the hearing threshold for any frequencies after AAV-ie injection, and the efficiencies of HC transfection in the apical, middle, and basal turns of the cochlea were 94.587 ± 1.381%, 93.150 ± 1.370%, and 86.320 ± 2.228%, respectively (Supplementary Fig. 2A and B). Cell counting verified that there was no significant difference in HC number between the AAV-ie group and the normal control group (Supplementary Fig. 2C and D). Western blot showed that the protein level of c-Myb in cochlear HCs in the AAV-c-Myb group was significantly higher than the normal control group (Supplementary Fig. 2E). Collectively, these results confirmed AAV-c-Myb transfection can effectively upregulate the expression of c-Myb in HCs in vivo without impairing auditory function or the cochlear HC number.

**Fig. 5:**
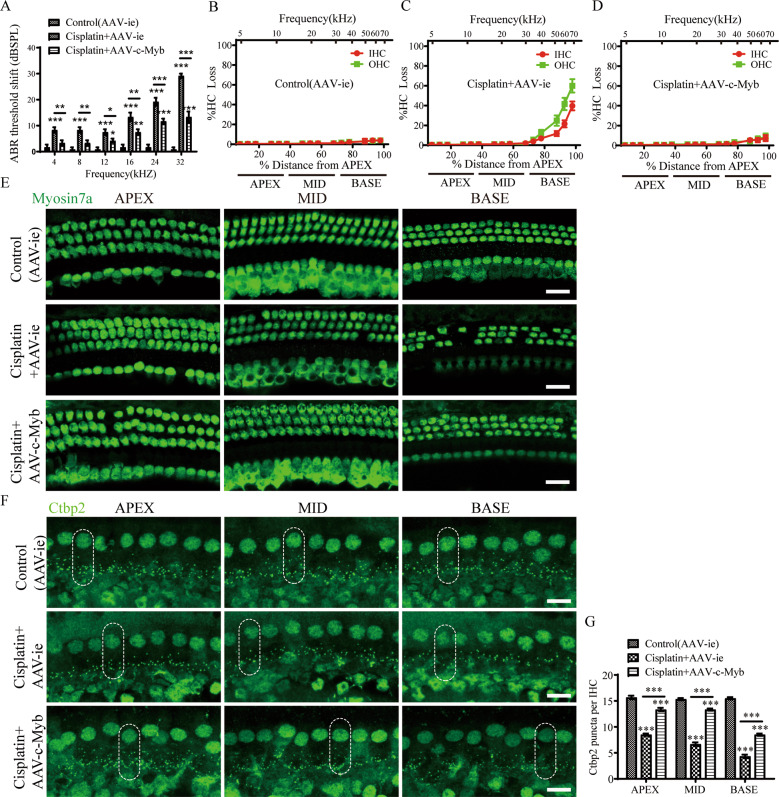
Overexpression of c-Myb in cochlear HCs in vivo improves HC survival and hearing function, protects ribbon synapse in mice after cisplatin injury. **A** The ABR threshold shifts at all frequencies from 4 to 32 were significantly reduced in the cisplatin + AAV-c-Myb compared to those in the cisplatin + AAV-ie group. **B**–**E** The immunostaining results and cochleogram showed that cochlear HCs pre-treated with AAV-c-Myb reduced the loss of HCs in the basal turns compared to cisplatin + AAV-ie group, but no significant difference of HC loss in the apial and middle turns of cochlea was found in cisplatin + AAV-c-Myb group compared with the cisplatin + AAV-ie group. Scale bar = 20 μm. **F**, **G** The average number of Ctbp2 puncta per IHC was significantly reduced in apex, middle and base turns of the cochlea after cisplatin treatment compared to the vehicle control, and the c-Myb overexpression by AAV-c-Myb protected against the loss of Ctbp2 puncta per IHC in all three turns of cochlea after cisplatin administration. Scale bar = 10 μm. ***p < 0.001, *n* = 6 for each group.

Then the role of c-Myb in cochlear HC survival and auditory function after cisplatin exposure in vivo was determined. The ABR threshold shifts at all frequencies from 4-32KHz were significantly reduced in the cisplatin + AAV-c-Myb compared to those in the cisplatin + AAV-ie group (Fig. 5A), and the loss of HCs was significantly reduced in the basal turns, but no significant difference of HC loss in the apical and middle turns was found in cisplatin + AAV-c-Myb group compared with the cisplatin + AAV-ie group (Fig. 5B–E, Supplementary Table 1). To address this distinct discrepancy between ABR changes and HC loss improvements in the apical and middle turns observed in vivo, we further analyzed the integrity of IHC ribbon synapses using the presynaptic marker, C-terminal binding protein 2 (Ctbp2). Results showed that the average number of Ctbp2 puncta per IHC was significantly reduced in apex, middle and base turns of the cochlea after cisplatin treatment compared to the vehicle control, and the c-Myb overexpression by AAV-c-Myb protected against the loss of Ctbp2 puncta per IHC in all three turns of cochlea after cisplatin administration (Fig. 5F and Supplementary Table 3). Together, these results suggest that overexpression of c-Myb in cochlear HCs in vivo promoted HC survival and protected hearing function, protects ribbon synapse after the mice were exposed to cisplatin.

### AAV-c-Myb transfection reduces apoptosis in cochlear HCs caused by cisplatin exposure in vivo

**Fig. 6:**
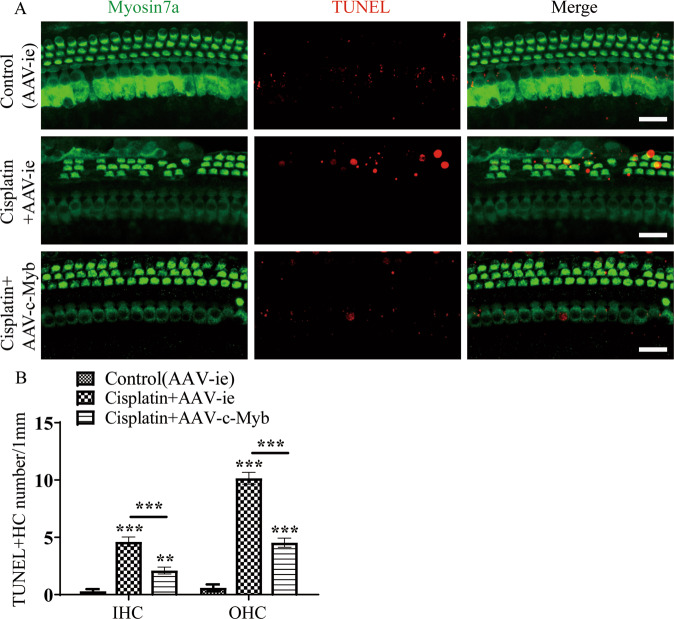
Overexpression of c-Myb in vivo inhibits cisplatin-induced cochlear HC apoptosis. **A**, **B** Representative images of TUNEL staining and cell counting showing that the numbers of TUNEL/Myosin7a double-positive IHCs and OHCs in the basal turns were increased significantly after cisplatin exposure in vivo compared to the vehicle control group, while the transfection of AAV-c-Myb led to a significantly reduced number of TUNEL/myosin7a double-positive HCs in the cisplatin + AAV-c-Myb group compared to the cisplatin + AAV-ie group. Scale bar = 20 μm. **p < 0.01, ***p < 0.001, *n* = 6 for each group.

The apoptosis in cochlear HCs were determined after the mice were treated with AAV-c-Myb and cisplatin in vivo as described above. As illustrated in Fig. 6, the number of TUNEL/myosin7a double-positive HCs in the cochlear basal turn was increased significantly after cisplatin exposure in adult mice compared to the vehicle control group, while the transfection of AAV-c-Myb led to a significantly reduced number of TUNEL/myosin7a double-positive HCs in the cisplatin + AAV-c-Myb group compared with the cisplatin + AAV-ie group (Fig. 6A and B, Supplementary Table 3). These results suggest that the overexpression of c-Myb in HCs inhibited cisplatin-induced cochlear HC apoptosis in vivo.

### c-Myb deficiency in OHCs exacerbated cisplatin-induced OHCs loss, apoptosis and hearing deficit in vivo

**Fig. 7:**
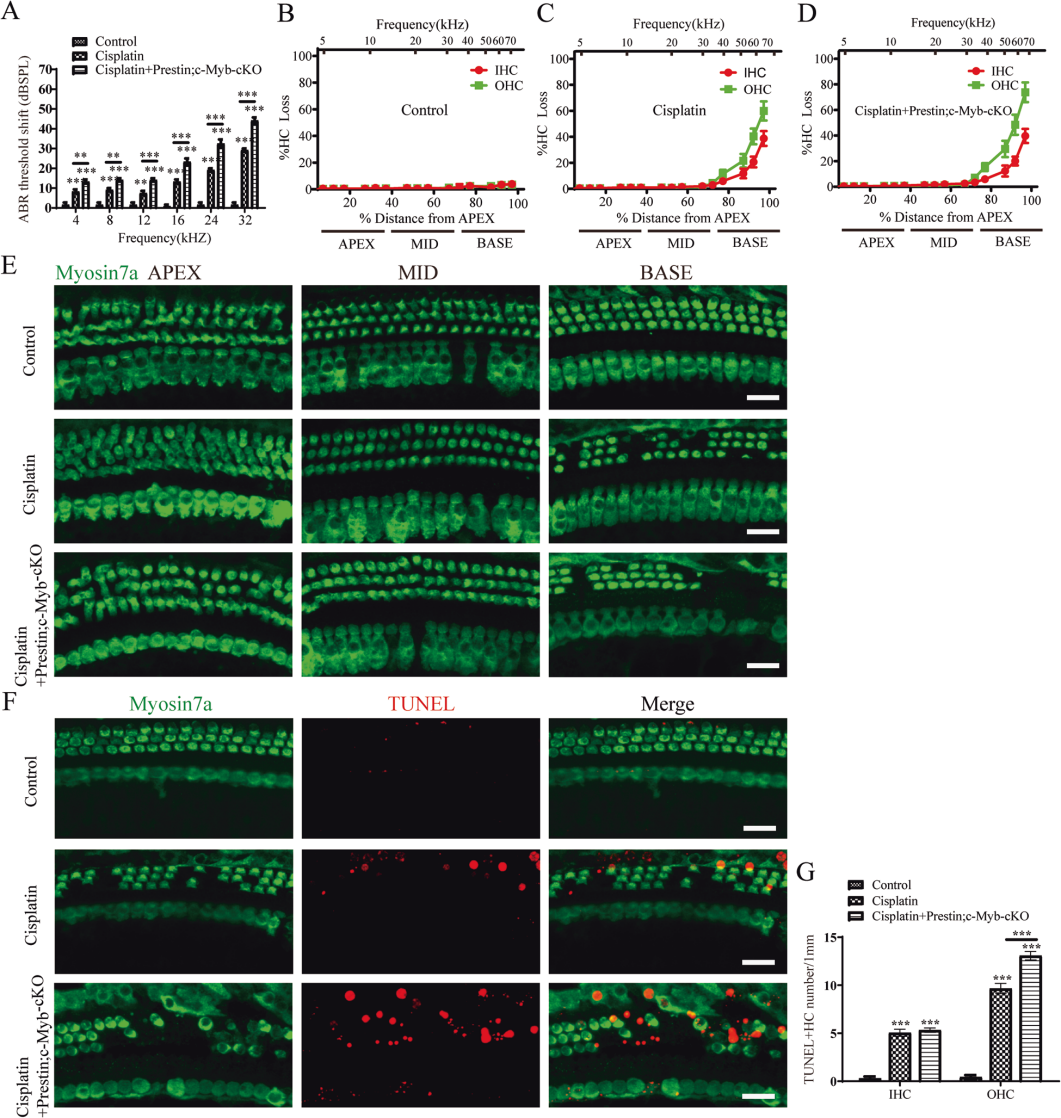
c-Myb deficiency in OHCs exacerbated cisplatin-induced OHCs loss, apoptosis and hearing deficit in vivo. **A** ABR threshold shifts at all frequencies from 4 to 32 kHz were significantly increased in the cisplatin + Prestin; c-Myb-cKO mice compared to those in the cisplatin group. **B**–**E** A significant increased OHC loss was found within the Prestin; c-Myb-cKO mice treated with cisplatin compared to the WT mice treated with cisplatin. **F**, **G** Basal turns of cochlear HCs were used for apoptosis analysis, and greater number of TUNEL/myosin7a double-positive OHCs were discovered in cisplatin + Prestin; c-Myb-cKO group compared to cisplatin group. Scale bar = 20 μm. **p < 0.01, ***p < 0.001, *n* = 6 for each group.

To further confirm the protection role of c-Myb in HC damage induced by cisplatin, c-Myb-HC conditional knockout mice (Prestin; c-Myb-cKO) in which c-Myb expression is downregulated only in cochlear OHCs were generated by crossing c-Myb flox/flox mice with Prestin-CreER mice. In this study, tamoxifen was injected in mice at P10–P12, and the cochlea were harvest at P30. Immunostaining showed that the c-Myb expression was significantly downregulated in OHCs but not in IHCs in Prestin; c-Myb-cKO mice at P30. Hearing test and cell counting showed that no statistical differences of hearing function and HC numbers were found between the Prestin; c-Myb-cKO mice and control mice (Supplementary Fig. 3), suggesting that the expression of c-Myb was efficiently downregulated in OHCs without affecting the hearing function and HC number in Prestin; c-Myb-cKO mice. Then the Prestin; c-Myb-cKO mice were injected with 3 mg/kg cisplatin from P 30 for 7 consecutive days, and the ABR threshold shifts at all frequencies from 4 to 32 kHz were significantly increased in the cisplatin + Prestin; c-Myb-cKO mice compared to those in the cisplatin group (Fig. 7A). A significant increased OHC loss was found within the Prestin; c-Myb-cKO mice treated with cisplatin compared to the WT mice treated with cisplatin (Fig. 7B–E). Basal turns of cochlear HCs were used for apoptosis analysis as described above, and greater number of TUNEL/myosin7a double-positive OHCs were discovered in cisplatin + Prestin; c-Myb-cKO group compared to cisplatin group (Fig. 7F and G). Together, these results suggest that c-Myb deficiency in OHCs exacerbated cisplatin-induced OHCs loss, apoptosis and hearing deficit in vivo.

### Overexpression of c-Myb reduces the cisplatin-induced damage to cochlear HCs at least partially by activating the PI3K/Akt signaling pathway in HCs

**Fig. 8:**
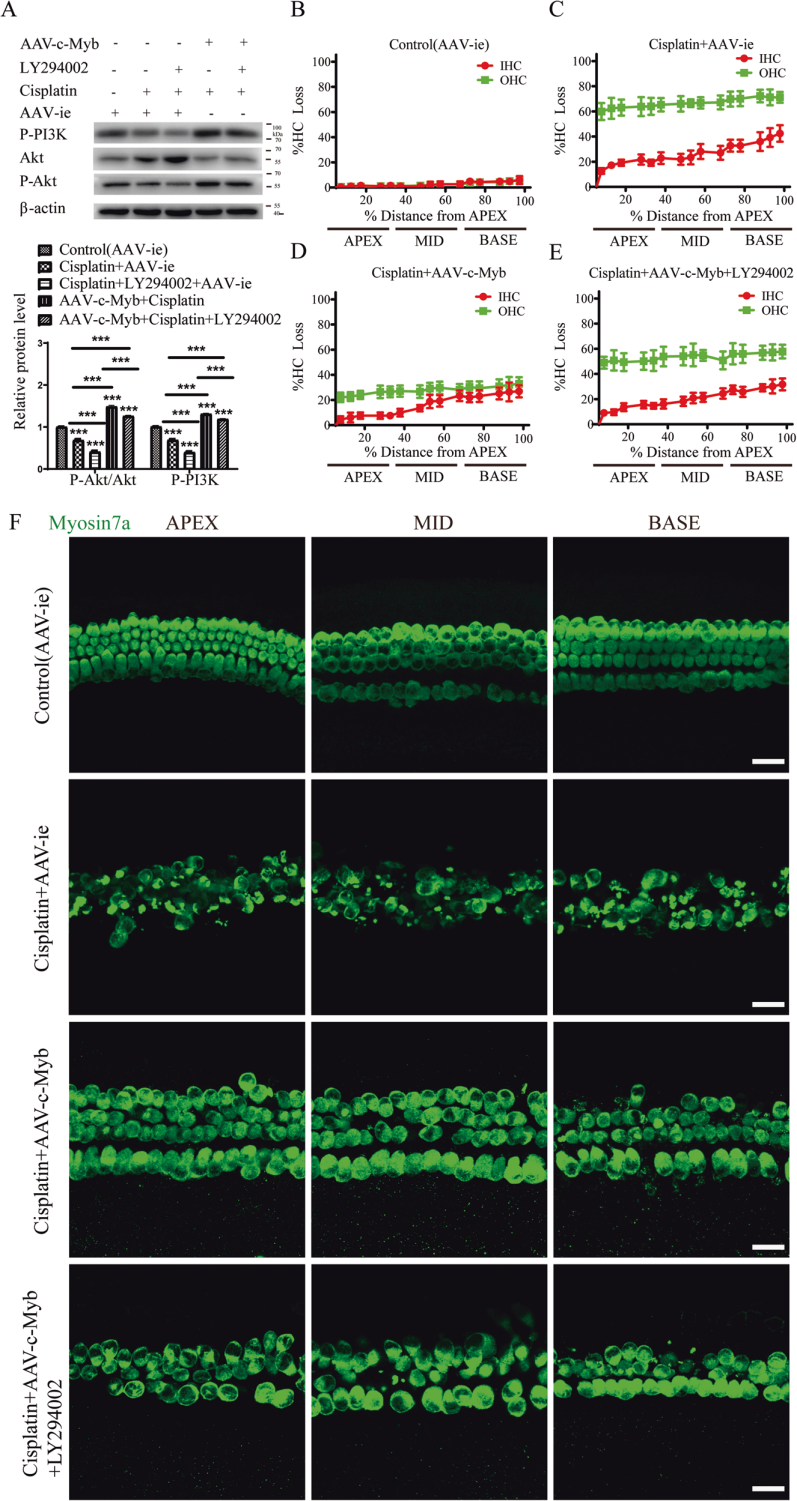
Upregulation of c-Myb reduces the cochlear HC damage induced by cisplatin by activating the PI3K/Akt signaling pathway. **A** Western blot showed a significantly decreased ratio of P-Akt/Akt and decreased level of P-PI3K (p85α) in the cultured HCs treated with cisplatin + AAV-ie for 48 h. The levels of P-PI3K (p85α) and P-Akt were significantly reduced in HCs co-treated with cisplatin, AAV-ie and LY294002 compared to the cisplatin + AAV-ie group, while they were significantly increased in HCs co-treated with AAV-c-Myb and cisplatin. The treatment with LY294002 decreased the above effects of c-Myb on p85α level and the ratio of P-Akt/Akt in the cisplatin + AAV-c-Myb + LY294002 group compared to the cisplatin + AAV-c-Myb group. **B**–**F** Immunostaining and cell counting showed that the overexpression of c-Myb reduced HC loss after cisplatin injury, but the treatment with LY294002 significantly increased the HC loss in the cisplatin + AAV-c-Myb + LY294002 group compared to the cisplatin + AAV-c-Myb group. Scale bar = 20 μm. ***p < 0.001, *n* = 6 for each group.

To address the mechanism mediating the c-Myb-dependent protection of HCs against cisplatin, we investigated the anti-apoptotic cell signaling pathway PI3K/Akt. The cultured cochlear HCs were cultivated in the presence of cisplatin and AAV-c-Myb with or without LY294002, a specific cell-permeable inhibitor of PI3K. We observed a significantly decreased ratio of phosphorylated (P)-Akt/Akt and decreased level of P-PI3K (p85α) in the cultured HCs treated with cisplatin + AAV-ie for 48 h (Fig. 8A). The levels of P-PI3K (p85α) and P-Akt were significantly reduced in cisplatin + AAV-ie + LY294002 group compared to the cisplatin + AAV-ie group. In contrast, the protein expression of p85α and the ratio of P-Akt/Akt was significantly increased in HCs co-treated with AAV-c-Myb and cisplatin, while the treatment with LY294002 decreased the above effects of c-Myb on p85α level and the ratio of P-Akt/Akt in the cisplatin + AAV-c-Myb + LY294002 group compared to the AAV-c-Myb + cisplatin group (Fig. 8A). Then, cell viability was determined and the overexpression of c-Myb reduced HC loss after cisplatin injury, but the treatment with LY294002 significantly increased the HC loss in the cisplatin + AAV-c-Myb + LY294002 group compared to the cisplatin + AAV-c-Myb group (Fig. 8B–F and Supplementary Table 4). The LY294002 treatment alone without cisplatin did not cause significant differences in HC number (Supplementary Fig. 4A and B). These results suggested that the activation of PI3K/Akt pathway might be regulated by transfection of exogenous c-Myb into HCs after cisplatin injury in vitro, and the inhibition of PI3K/Akt signaling pathway abolished the effect of c-Myb on improving HC survival after cisplatin damage.

## Discussion

Our previous work showed that c-Myb is specifically expressed in cochlear HCs after birth and that c-Myb knockdown exacerbates the neomycin-induced damage to HEI-OC1 cells in vitro [21]. Despite that report, the function of c-Myb in the mammalian inner ear, as well as its underlying mechanisms, remain poorly understood. In the current study, we show for the first time that the expression of c-Myb in the cochlear HCs of adult mice was significantly decreased after cisplatin treatment (Fig. 1), this finding is consistent with our previous discovery that c-Myb expression is reduced in the surviving HCs after neomycin treatment in vivo [21] and thus indicates that c-Myb might also play a role against cisplatin-induced HC damage.

In this study, the role of c-Myb in HCs treated with cisplatin was clarified by performing functional studies via overexpressing or knocking down c-Myb in HCs. We found that the cisplatin-induced HC loss and apoptosis in the basal turn cochlea were significantly improved both in vitro and in vivo after c-Myb overexpression, while they were exacerbated in the c-Myb-deficient HCs of basal turn cochlea in vivo. Studies have reported that c-Myb is involved in cell survival through the regulation of numerous target genes, and knockout of c-Myb is significantly related to cell death [14, 27]. The inhibition of c-Myb induces apoptosis in U937 and K562 cells [28], and that c-Myb attenuates cell viability and increases apoptosis in SH-SY5Y cells [17] and aggravates neomycin-induced apoptosis in HEI-OC1 cells [21]. The findings in this study also verified that c-Myb is required for the prevention of programmed cell death in HCs induced by the ototoxic drug cisplatin.

We noticed that no significant HC loss was found in the apex and middle turns of cochlea induced by cisplatin, in spite of hearing thresholds elevation was occurred at lower to middle frequencies. Moreover, c-Myb protected against cisplatin-induced hearing deficit at all frequencies tested, even though significant increase of HC number was restricted to the basal region of the cochlea. Thus, it seems that there were other pathological changes in apical and middle cochlear turns. One possible explanation is that as there are three main targets of cisplatin in cochlea, i.e. HCs, stria vascularis and SGNs, the damage caused by cisplatin to other cells, except for HCs, in all three turns of cochlea can contribute to ABR threshold shift in all frequencies. For example, Alam et al. demonstrated TUNEL-positive cells was significantly induced by cisplatin in stria vascularis of all three turns, while TUNEL-positive HCs and SGNs were only found in the basal turns [29]. Another reason for the apparent discrepancy between hearing loss and lack of HC loss in the apical and middle turns observed in vivo might be the reduction of ribbon synapses. The ribbon synapses are intimately involved in IHC neurotransmission via regulating vesicular release of neurotransmitters. It has been reported that IHC ribbon synapses are more susceptible to ototoxic drugs or noise stimulation than the other cochlear components included OHCs, IHCs, and SGNs [30–32]. Our results showed that significant reductions in the average number of Ctbp2 puncta per IHC were found in the apical, middle and basal turns after cisplatin damage, and c-Myb overexpression increased the number of Ctbp2 puncta in all turns of cochlea treated with cisplatin. In view of the importance of Ctbp2 and other ribbon synaptic proteins for neurotransmission in IHCs, we believe that the ability of c-Myb to maintain the levels of Ctbp2 in IHCs could contribute to its overall efficacy as an otoprotective agent.

It has been documented that increased ROS production is a major mechanism underlying cisplatin-induced HC death [33]. Recent studies have shown that the enhanced activity of NADPH oxidase (NOX), including the isoforms NOX1, NOX3 and NOX4, and the interference with the antioxidant defense system are the main sources of ROS generation employed by cisplatin in the cochlea [34, 35]. In this study, c-Myb overexpression significantly reduced the level of ROS in HCs after cisplatin exposure (Fig. 4), suggesting that c-Myb exerts its protective functions against cisplatin by alleviating oxidative stress in HCs. A previous study identified that NOX1 might be a c-Myb-targeted gene and that the NOX1 activity is required for the c-Myb-mediated cell protection from cytotoxic agents [36]. Given that the inhibition of NOX-1 and NOX-4 expression markedly abolished the cytotoxicity and ROS generation in auditory cells induced by cisplatin [37], the mechanism underlying the cytoprotection of c-Myb in which c-Myb regulate NOX1 expression and subsequently alleviates ROS accumulation in HCs after cisplatin injury is worthy to be explored in the future study.

PI3K/Akt is an important signaling pathway regulating various cellular processes such as cell survival, apoptosis, metabolism, and proliferation. Furthermore, the PI3K/Akt signaling pathway is involved in mediating the survival of sensory HCs, and studies have shown that activation of PI3K-Akt signaling can reduce the HC loss induced by gentamicin [38], protect HCs from TNF-α-induced cell death to prevent hearing loss [39], promote the survival of supporting cells [40], and protect SGNs [11, 41]. In addition, Lee et al. reported that c-Myb mediates activation of p-Akt and that c-Myb promotes cell survival via p-AMPK/Akt signaling under conditions of glucose oxidative stress in dental pulp cells [42]. In this study, our finding that the inhibition of PI3K/Akt pathway reverses the protective effect of c-Myb against HC loss after cisplatin damage is consistent with the previous studies, which proved that the inhibition of PI3K increased HC sensitivity to noise or ototoxic drugs damage [43–45]. Nevertheless, the specific regulation mechanisms of c-Myb on PI3K/Akt signaling pathway in HCs was not directly addressed in this study, and they can be complex. Kim et al. reported that c-Myb gene might stimulate leukemia cell growth by IGF-IR mediated Akt signaling pathway [46]. Further characterization of the interaction between c-Myb and the PI3K-Akt pathway and their effects on hearing protection will possibly contribute to the development of therapeutic interventions for hearing loss.

In summary, we determined the mechanistic details behind the neuroprotective effect of c-Myb in cochlear HCs against cisplatin injury. We found that c-Myb could strongly promote HC survival, decrease HC apoptosis, and reduce intracellular ROS levels after cisplatin exposure in vitro and in vivo as well as improve hearing function of mice after cisplatin injury. Moreover, we showed that this protective mechanism involves activation of the PI3K/Akt signaling pathway in HCs exposed to cisplatin. The findings from this work suggest that c-Myb might serve as a novel target for the prevention of cisplatin-induced HC damage and hearing loss.

## Materials and methods

### Animals and drug treatments

C57BL/6 J mice were purchased from the Animal Center of Shandong University (Jinan, China). The c-Myb conditional knockout mice (c-Myb flox/flox) in the C57BL/6 background were bought from Cyagen Biosciences Inc. (No. CKOCMP-17863-Myb-B6N-VA, Taicang, China), Prestin-CreER mice [47] were gifts from Prof. Zhiyong Liu (Chinese Academy of Sciences, Shanghai, China). The c-Myb-HC specific knockout mice (Prestin; c-Myb-cKO) in which c-Myb expression is downregulated only in cochlear OHCs were generated by crossing c-Myb flox/flox mice with Prestin-CreER mice. The Cre activity in Prestin-CreER mice was induced with tamoxifen at various postnatal ages and only detected in OHCs [47], resembling the endogenous prestin expression pattern. Tamoxifen (3 mg/40 g body weight, intraperitoneal injection; Sigma) was injected once daily at P10-P12 to induce Cre activity. The genotyping for all transgenic mice with PCR was performed using genomic DNA from tail tips incubated in 70 μL 50 mM NaOH at 98 °C for 1 h, followed by neutralization in 7 μL 1 M HCl. The genotyping primers are: Prestin F 5′-CACAAGTTGTGAATGACCTC-3′, 5′-GTTAAAGAGCGTAATCTGGAACA-3′, and Prestin R 5′-TAACTGCTAGCATTTC CCTT-3′, c-Myb F: 5′-CCAATGTTTGGTAAACACTTTGGCT-3′, c-Myb R: 5′-ACAACTCCACTTAACAAACTCACTG-3′. All animal procedures were performed according to protocols approved by the Animal Care Committee of Shandong University, China (No. ECAESDUSM 20123011) and were consistent with the National Institute of Health’s Guide for the Care and Use of Laboratory Animals. All animals were bred and housed in a climate-controlled room with an ambient temperature of 23 ± 2 °C and a 12/12 h light/dark cycle.

To establish animal models of hearing loss induced by cisplatin in vivo, age and sex-matched mice were randomly divided into experimental and control groups, experimental mice were given a daily i.p. injection of cisplatin (cisplatin group, 3 mg/kg) and control mice were given 0.9% sterile saline (control group, 0.06 ml/kg) starting from P 30 for 7 consecutive days. Mice in the cisplatin + AAV-c-Myb group were injected with 1 × 10^10^ GCs of AAV-c-Myb into the left scala tympani through the left ear RWM, as described in the following Animal surgery section, at P16 and then i.p. injected as above with 3 mg/kg cisplatin from P30 to P36. Mice in the control (AAV-ie) and cisplatin + AAV-ie group were injected with 1 × 10^10^ GCs of AAV-ie vector into the left scala tympani through the left ear RWM at P16 and then i.p. injected as above with 0.9% sterile saline or 3 mg/kg cisplatin from P30 to P36.

### Virus construction

The c-Myb-overexpressing AAV was constructed with the AAV vector AAV-ie containing the mouse *c-Myb* gene. Briefly, the C-terminal FLAG-tagged NLS-mNeonGreen was cloned into the AAV-ie plasmid containing the cytomegalovirus enhancer/chicken β-actin (CAG) promoter and the woodchuck hepatitis virus post-transcriptional regulatory element (WPRE) cassette that was flanked by AAV2 inverted terminal repeats. The AAV-ie serotype vector was produced in HEK 293T cells co-transfected with RepCap-fused plasmid and a helper plasmid. The AAV-ie carrying the coding sequence of *c-Myb* driven by the CAG promoter was generated and named AAV-c-Myb. The virus was purified by iodixanol gradient ultra-centrifugation with a titer of 3 × 10^13^ GCs/mL. Titers were calculated by qPCR with WPRE primers as follows: forward, 5′-GTCAGGCAACGTGGCGTGGTGTG-3′; reverse, 5′-GGCGATGAGTTCCGCCG TGGC-3′. Virus aliquots were stored at –80 °C, and the efficiencies of virus transfection and c-Myb expression were evaluated first as described in the text.

### Quantification of green fluorescent protein (mNeonGreen) expression

The efficiency of virus transfection in HCs was determined by the quantification of green fluorescent protein (mNeonGreen) expression. For the efficiency of virus transfection in vitro, the cultured cochlear HCs from P3 mice were transfected with AAV-ie at 2 × 10^10^ genome-containing particles (GCs) per tissue sample for 60 h. For the efficiency of virus transfection the in vivo, the mice were injected with 1 × 10^10^ GCs of AAV-ie through the round window membrane (RWM) at P16, and the mice were sacrificed after ABR measurements at P30. After the immunostaining, cochlear HCs were co-labeled with anti-myosin 7a and mNeonGreen, and ×80 magnification images were taken of the apical, middle, and basal turns of the cochlea, and the number of mNeonGreen-expressing cochlear HCs was counted and averaged for every 100 μm along the cochlea. The infection efficiencies of the apical, middle, and basal turns of each cochlea were calculated separately.

### Organotypic culture of neonatal mouse cochlear HCs and drug treatments in vitro

The organotypic culture of neonatal mouse cochlear HCs followed our previously published procedure [48]. Briefly, P3 C57BL/6 mice were decapitated and their skulls were opened along the midline of the sagittal suture of the skull with surgical scissors. The temporal bones on both sides were dissected and placed into sterile Hank’s Balanced Salt Solution (Hyclone, USA) on a flat ice pack. The cochlear capsule was removed by fine forceps under a dissecting microscope to expose the membranous labyrinth, and the stria vascularis and modiolus were removed. Wholemount cochleae were then placed onto 10 mm coverslips (Fisher Scientific, USA) pre-coated with CellTaK (BD Biosciences, USA) and incubated in Dulbecco’s Modified Eagle Medium/F12 (DMEM/F12, Invitrogen, USA) supplemented with 1% fetal bovine serum,1% N2, and 50 mg/ml ampicillin (Sigma, USA) at 37 °C in a 5% CO_2_ atmosphere.

The virus treatment was based on our previously published article [21] with modification. In the cisplatin + AAV-c-Myb group, the cultured HCs were pretreated with 2 × 10^10^ GCs of AAV-c-Myb for 12 h and then continued to be co-treated with 30 μM [23] cisplatin for 48 h. In the control (AAV-ie) and cisplatin + AAV-ie group, 2 × 10^10^ GCs of AAV-ie vector were pre-added into culture media for 12 h and then continued to be co-treated with or without 30 μM cisplatin for 48 h. In the cisplatin + AAV-c-Myb + LY294002 group, the cultured HCs were pretreated with 2 × 10^10^ GCs of AAV-c-Myb for 12 h, pretreated with LY294002 (10 μM; Cell Signaling Technology Inc, USA) for 2 h, and then continued to be co-treated with 30 μM cisplatin for 48 h.

### Animal surgery

Virus injection through the RWM was performed as described previously [22] with modification. Briefly, P16 C57BL/6J mice were anesthetized with ketamine (50 mg/kg, intramuscular injection) and xylazine (6 mg/kg, intramuscular injection). Each mouse was operated on the left ear, and the right ear was used as a control. A post-auricular incision was made, and the tissue was dissected to open the otic bulla and expose the round window niche. Injections were performed through the RWM with a glass micropipette (25 μm) controlled by a micromanipulator UMP3 UltraMicroPump (World Precision Instruments). The volume of the injected virus (AAV-ie or AAV-c-Myb) was ~0.33 μL per cochlea over 120 s. After the injection, the skin incision was sealed using veterinary tissue adhesive (Millpledge Ltd, UK). Mice were resuscitated in a 30 °C incubator.

### Immunostaining

For cultured cochleae, the basilar membranes were fixed in 4% paraformaldehyde. For adult mouse cochleae, cochlear samples were fixed in 4% paraformaldehyde overnight and then decalcified with 0.5 mM EDTA (pH 8.0) for 24 h and then the basilar membranes were cut from the apex to the base and placed onto 10 mm coverslips pre-coated with Cell-Tak. Samples from both cultured cochlea and adult mice were then permeabilized with 1% Triton X-100 (Sigma-Aldrich) in PBS and blocked with 8% donkey serum in 0.1% Triton X-100, 1% bovine serum albumin, and 0.02% sodium azide dissolved in PBS at room temperature for 1 h. The samples were then stained with primary antibodies against myosin 7a (1:1000 dilution, Proteus Biosciences, and1:800 dilution, DSHB), c-Myb (1:1000 dilution, Millipore, USA), anti-cleaved-caspase3 (1:1000 dilution, Cell Signaling Technology Inc.), and anti-Ctbp2 (1:1000 dilution, BD Biosciences) overnight at 4 °C. The next day, the samples were incubated with secondary antibodies (1:1000 dilution, Invitrogen) along with DAPI (1:1000 dilution, Sigma-Aldrich) in 0.1% Triton X-100 and 1% bovine serum albumin in PBS at room temperature for 1 h. The coverslips were mounted and photographed under a laser scanning confocal microscope (Leica SP8; Leica, Germany).

### TUNEL

Apoptosis in HCs was measured with a TUNEL staining kit (Click-iT Plus TUNEL Assay for In Situ Apoptosis Detection; Invitrogen) according to the manufacturer’s instructions, and the samples were incubated with the anti-myosin 7a primary antibody. Finally, the coverslips were mounted and observed under a laser scanning confocal microscope (Leica SP8; Leica, Germany).

### ABR

ABR testing was used to measure the auditory function of adult mice. The mice were anesthetized with ketamine (50 mg/kg, intramuscular injection) and xylazine (6 mg/kg, intramuscular injection) and kept at 38 °C with a thermal static heating device (FHC, USA). The non-inverting electrode was inserted into the apex of the skull, and the reference electrode and ground electrode were inserted into the neck behind the auditory bullae on both sides. Changes in brain electrical activity caused by tone bursts were recorded by the electrodes. ABR responses were triggered in tone bursts at 4, 8, 12, 16, 24, and 32 kHz. The TDT System III (Tucker-Davis Technologies, USA) hardware and software were used to send stimuli and record the responses, with 1,024 stimuli repetitions for each recording. The sound level was decreased from 90 to 0 dB sound pressure level (decibels SPL) in 5 dB steps. The lowest audible SPL that produced a response was recorded as the hearing threshold.

### Western blot

For the groups cultured in vitro, after removing the culture medium the basilar membranes were washed three times with PBS. For the in vivo treatment groups, the mice were sacrificed by cervical dislocation, the temporal bones were dissected, the cochlear bones were carefully peeled off, and the basilar membranes were washed three times with PBS. The basilar membranes were added to radio-immunoprecipitation assay lysis buffer (Sigma-Aldrich, R0278) and lysed for 30 min on ice, then the supernatant was collected. The protein concentration was determined by the BCA assay (P0012, Beyotime Institute of Biotechnology). A total of 30 µg of each protein sample was denatured and separated by 10% SDS-PAGE and then transferred to a polyvinylidene fluoride membrane and blocked with 5% non-fat dried milk in Tris-buffered saline and Tween 20 (TBST) for 1 h at room temperature. The primary antibodies were as follows: anti-c-Myb antibody (1:1000 dilution, Millipore, USA), anti-P-Akt (1:1000 dilution; Cell Signaling Technology), anti-Akt (1:1000 dilution; Cell Signaling Technology), anti-P-PI3K (1:1000 dilution; Cell Signaling Technology), and anti-β-actin (1:2000 dilution; ZSGB-BIO). The corresponding secondary antibodies were then added. Semi-quantification of the western blot results was performed using ImageJ software to measure the intensities of the bands. The band densities were normalized to background, and then the relative optical density ratio was calculated by comparison to β-actin. Full uncropped scans of original gels were displayed in supplementary materials (Uncropped gels of western blot).

### Hair cell counting and cochleogram plotting

The quantitative evaluation of HCs was performed according to the previously published report [48]. Briefly, after immunostaining the HCs with myosin 7a, the apical, middle, and basal turns were first imaged under ×20 magnification to identify regions of interest, and at least two Z-stacks from non-overlapping regions were then obtained for each turn using a ×40 objective. Image J software (NIH) was used to measure the length of the selected cochlear segments and the total number of HCs was counted. Then the percent loss of hair cells for each 0.247 mm segment in vitro and each 0.300 mm segment in vivo was calculated. Each 0.247 or 0.300 mm segment represents a certain percentage of the cochlear length depending on the total length. To make an average of the hair cell loss for cochleae of different lengths, the mm segments were converted into percent corresponding to equal frequency steps (4, 8, 12, etc) for each cochlea. The frequency-place equation by Müller et al. [49] was used to correlate the location to frequency.

### Statistical analysis

All data are shown as the mean or mean ± SEM, and all experiments were repeated more than three times. Statistical analyses were carried out using the SPSS 21 software. Two-tailed, unpaired Student’s *t*-tests were used to determine statistical significance when comparing two groups. A one-way ANOVA followed by a Dunnett’s multiple comparisons test was used when comparing more than two groups. A value of *p* < 0.05 was considered statistically significant. Scale bars and *n* values are defined in the respective figures and legends and *n* represents the number of independent cochlear samples from each sub-group.

## Supplementary information

Supplementary figures

Supplementary tables

uncropped western blots

## Data Availability

The datasets used and/or analyzed during the current study are available from the corresponding author on reasonable request.

## References

[1] Tanna RJ, Lin JW, De Jesus O. Sensorineural Hearing Loss. StatPearls. Treasure Island (FL) 2021. 33351419

[2] Dasari S, Tchounwou PB (2014). Cisplatin in cancer therapy: molecular mechanisms of action. Eur. J. Pharmacol..

[3] Barabas K, Milner R, Lurie D, Adin C (2008). Cisplatin: a review of toxicities and therapeutic applications. Vet. Comp. Oncol..

[4] Freyer DR, Brock PR, Chang KW, Dupuis LL, Epelman S, Knight K (2020). Prevention of cisplatin-induced ototoxicity in children and adolescents with cancer: a clinical practice guideline. Lancet Child Adolesc. Health.

[5] van Ruijven MW, de Groot JC, Klis SF, Smoorenburg GF (2005). The cochlear targets of cisplatin: an electrophysiological and morphological time-sequence study. Hearing Res..

[6] van Ruijven MW, de Groot JC, Smoorenburg GF (2004). Time sequence of degeneration pattern in the guinea pig cochlea during cisplatin administration. A quantitative histological study. Hearing Res..

[7] Rybak LP (2007). Mechanisms of cisplatin ototoxicity and progress in otoprotection. Curr Opin Otolaryngol Head Neck Surg..

[8] Gentilin E, Simoni E, Candito M, Cazzador D, Astolfi L (2019). Cisplatin-induced ototoxicity: updates on molecular targets. Trends Mol Med..

[9] Langer T, am Zehnhoff-Dinnesen A, Radtke S, Meitert J, Zolk O (2013). Understanding platinum-induced ototoxicity. Trends Pharmacol Sci..

[10] Liu W, Xu X, Fan Z, Sun G, Han Y, Zhang D (2019). Wnt signaling activates TP53-induced glycolysis and apoptosis regulator and protects against cisplatin-induced spiral ganglion neuron damage in the mouse cochlea. Antioxid Redox Signal..

[11] Liu W, Xu L, Wang X, Zhang D, Sun G, Wang M, et al. PRDX1 activates autophagy via the PTEN-AKT signaling pathway to protect against cisplatin-induced spiral ganglion neuron damage. Autophagy. 2021;17:4159–81. 10.1080/15548627.2021.1905466 PMC8726717 33749526

[12] Previati M, Lanzoni I, Astolfi L, Fagioli F, Vecchiati G, Pagnoni A (2007). Cisplatin cytotoxicity in organ of Corti-derived immortalized cells. J Cell Biochem..

[13] Zhang N, Cai J, Xu L, Wang H, Liu W (2020). Cisplatin-induced stria vascularis damage is associated with inflammation and fibrosis. Neural Plasticity.

[14] Mucenski ML, McLain K, Kier AB, Swerdlow SH, Schreiner CM, Miller TA (1991). A functional c-myb gene is required for normal murine fetal hepatic hematopoiesis. Cell..

[15] Vegiopoulos A, Garcia P, Emambokus N, Frampton J (2006). Coordination of erythropoiesis by the transcription factor c-Myb. Blood.

[16] Zubair H, Patel GK, Khan MA, Azim S, Zubair A, Singh S (2020). Proteomic analysis of MYB-regulated secretome identifies functional pathways and biomarkers: potential pathobiological and clinical implications. J. Proteome Res..

[17] Zhang J, Shu Y, Qu Y, Zhang L, Chu T, Zheng Y (2017). C-myb plays an essential role in the protective function of IGF-1 on cytotoxicity induced by Abeta25-35 via the PI3K/Akt pathway. J Mol Neurosci..

[18] Tian M, Tian D, Qiao X, Li J, Zhang L (2019). Modulation of Myb-induced NF-kB -STAT3 signaling and resulting cisplatin resistance in ovarian cancer by dietary factors. J Cell Physiol..

[19] Azim S, Zubair H, Srivastava SK, Bhardwaj A, Zubair A, Ahmad A (2016). Deep sequencing and in silico analyses identify MYB-regulated gene networks and signaling pathways in pancreatic cancer. Sci Rep..

[20] Betancur P, Sauka-Spengler T, Bronner M (2011). A Sox10 enhancer element common to the otic placode and neural crest is activated by tissue-specific paralogs. Development..

[21] Yu X, Liu W, Fan Z, Qian F, Zhang D, Han Y (2017). c-Myb knockdown increases the neomycin-induced damage to hair-cell-like HEI-OC1 cells in vitro. Sci Rep..

[22] Tan F, Chu C, Qi J, Li W, You D, Li K (2019). AAV-ie enables safe and efficient gene transfer to inner ear cells. Nat Commun..

[23] Yang Q, Sun G, Yin H, Li H, Cao Z, Wang J (2018). PINK1 Protects auditory hair cells and spiral ganglion neurons from cisplatin-induced ototoxicity via inducing autophagy and inhibiting JNK signaling pathway. Free Radic Biol Med..

[24] Ding D, He J, Allman BL, Yu D, Jiang H, Seigel GM (2011). Cisplatin ototoxicity in rat cochlear organotypic cultures. Hearing Res..

[25] Cardinaal RM, de Groot JC, Huizing EH, Veldman JE, Smoorenburg GF (2000). Dose-dependent effect of 8-day cisplatin administration upon the morphology of the albino guinea pig cochlea. Hear Res.

[26] Schellens JH, Planting AS, Ma J, Maliepaard M, de Vos A, de Boer Dennert M (2001). Adaptive intrapatient dose escalation of cisplatin in patients with advanced head and neck cancer. Anticancer Drugs.

[27] Oh IH, Reddy EP (1999). The myb gene family in cell growth, differentiation and apoptosis. Oncogene..

[28] Ye P, Zhao L, McGirr C, Gonda TJ (2014). MYB down-regulation enhances sensitivity of U937 myeloid leukemia cells to the histone deacetylase inhibitor LBH589 in vitro and in vivo. Cancer Lett..

[29] Alam SA, Ikeda K, Oshima T, Suzuki M, Kawase T, Kikuchi T (2000). Cisplatin-induced apoptotic cell death in Mongolian gerbil cochlea. Hearing Res..

[30] Borse V, Al Aameri RFH, Sheehan K, Sheth S, Kaur T, Mukherjea D (2017). Epigallocatechin-3-gallate, a prototypic chemopreventative agent for protection against cisplatin-based ototoxicity. Cell Death Dis..

[31] Liu K, Jiang X, Shi C, Shi L, Yang B, Shi L (2013). Cochlear inner hair cell ribbon synapse is the primary target of ototoxic aminoglycoside stimuli. Mol. Neurobiol..

[32] Shi L, Guo X, Shen P, Liu L, Tao S, Li X (2015). Noise-induced damage to ribbon synapses without permanent threshold shifts in neonatal mice. Neuroscience..

[33] Casares C, Ramirez-Camacho R, Trinidad A, Roldan A, Jorge E, Garcia-Berrocal JR (2012). Reactive oxygen species in apoptosis induced by cisplatin: review of physiopathological mechanisms in animal models. Eur Arch Otorhinolaryngol.

[34] Mukherjea D, Jajoo S, Kaur T, Sheehan KE, Ramkumar V, Rybak LP (2010). Transtympanic administration of short interfering (si)RNA for the NOX3 isoform of NADPH oxidase protects against cisplatin-induced hearing loss in the rat. Antioxid Redox Signal..

[35] Ravi R, Somani SM, Rybak LP (1995). Mechanism of cisplatin ototoxicity: antioxidant system. Pharmacol Toxicol..

[36] Pekarcikova L, Knopfova L, Benes P, Smarda J (2016). c-Myb regulates NOX1/p38 to control survival of colorectal carcinoma cells. Cell Signal..

[37] Kim HJ, Lee JH, Kim SJ, Oh GS, Moon HD, Kwon KB (2010). Roles of NADPH oxidases in cisplatin-induced reactive oxygen species generation and ototoxicity. J Neurosci..

[38] Kucharava K, Sekulic-Jablanovic M, Horvath L, Bodmer D, Petkovic V (2019). Pasireotide protects mammalian cochlear hair cells from gentamicin ototoxicity by activating the PI3K-Akt pathway. Cell Death Dis..

[39] Haake SM, Dinh CT, Chen S, Eshraghi AA, Van De Water TR (2009). Dexamethasone protects auditory hair cells against TNFalpha-initiated apoptosis via activation of PI3K/Akt and NFkappaB signaling. Hearing Res..

[40] Jadali A, Ying YM, Kwan KY (2017). Activation of CHK1 in supporting cells indirectly promotes hair cell survival. Front Cell Neurosci..

[41] Euteneuer S, Yang KH, Chavez E, Leichtle A, Loers G, Olshansky A (2013). Glial cell line-derived neurotrophic factor (GDNF) induces neuritogenesis in the cochlear spiral ganglion via neural cell adhesion molecule (NCAM). Mol Cell Neurosci..

[42] Lee YH, Kim HS, Kim JS, Yu MK, Cho SD, Jeon JG (2016). C-myb regulates autophagy for pulp vitality in glucose oxidative stress. J Dent Res..

[43] Chung WH, Pak K, Lin B, Webster N, Ryan AF (2006). A PI3K pathway mediates hair cell survival and opposes gentamicin toxicity in neonatal rat organ of Corti. J Assoc Res Otolaryngol..

[44] Ma W, Hu J, Cheng Y, Wang J, Zhang X, Xu M (2015). Ginkgolide B protects against cisplatin-induced ototoxicity: enhancement of Akt-Nrf2-HO-1 signaling and reduction of NADPH oxidase. Cancer Chemother Pharm..

[45] Chen J, Yuan H, Talaska AE, Hill K, Sha SH (2015). Increased sensitivity to noise-induced hearing loss by blockade of endogenous PI3K/Akt signaling. J. Assoc. Res Otolaryngol..

[46] Liu Y, Wei M, Mao X, Chen T, Lin P, Wang W (2021). Key signaling pathways regulate the development and survival of auditory hair cells. Neural Plasticity.

[47] Fang J, Zhang WC, Yamashita T, Gao J, Zhu MS, Zuo J (2012). Outer hair cell-specific prestin-CreERT2 knockin mouse lines. Genesis..

[48] Cao Z, Yang Q, Yin H, Qi Q, Li H, Sun G (2017). Peroxynitrite induces apoptosis of mouse cochlear hair cells via a Caspase-independent pathway in vitro. Apoptosis.

[49] Viberg A, Canlon B (2004). The guide to plotting a cochleogram. Hearing Res..

