# Supplementary material for: c-Myb protects cochlear hair cells from cisplatin-induced damage via the PI3K/Akt signaling pathway
Source: Cell Death Discov. 2022 Feb 24;8:78. doi: 10.1038/s41420-022-00879-9 (PMC8873213; doi:10.1038/s41420-022-00879-9)
Supplement: Supplementary file 1 — Supplementary figures [file 41420_2022_879_MOESM1_ESM.docx]

**Supplementary Figure**


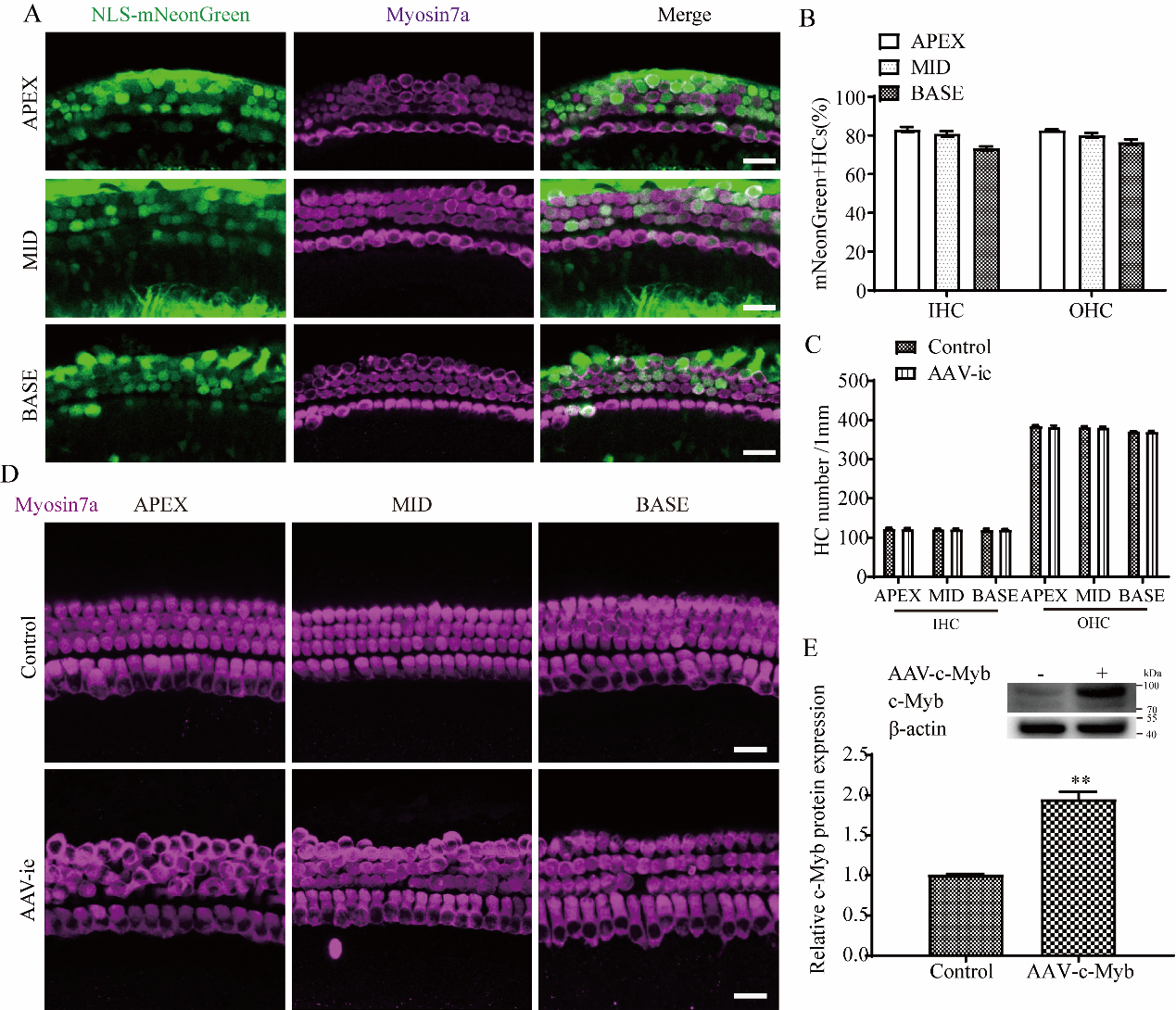


Supplementary Figure 1. **c-Myb is upregulated in cultured cochlear HCs after transfection with AAV-c-Myb. (A, B**) The cultured cochlear HCs from P3 mice were infected with 2 × 10^10^ GCs of AAV-ie for 60 h, and immunostaining by nuclear localization sequence-mNeonGreen (NLS-mNeonGreen) fluorescence (green) showed that 83.5 ± 1.2%, 80.1 ± 4.3%, and 77.4 ± 3.3% of HCs (Myosin 7a, purple) in the apical, middle, and basal turns were positively transfected, respectively. Scale bar = 20µm. (**C**, **D**) The number counting of HCs showed that there was no significant difference in HC number between the AAV-ie group and the control group. (**E)** Western blot showed that after 2 × 10^10^ GCs of AAV-c-Myb were transfected into the cultured cochlear HCs for 60 h the level of c-Myb protein was increased significantly compared with the control group. n =3 for each group, *p<0.05.


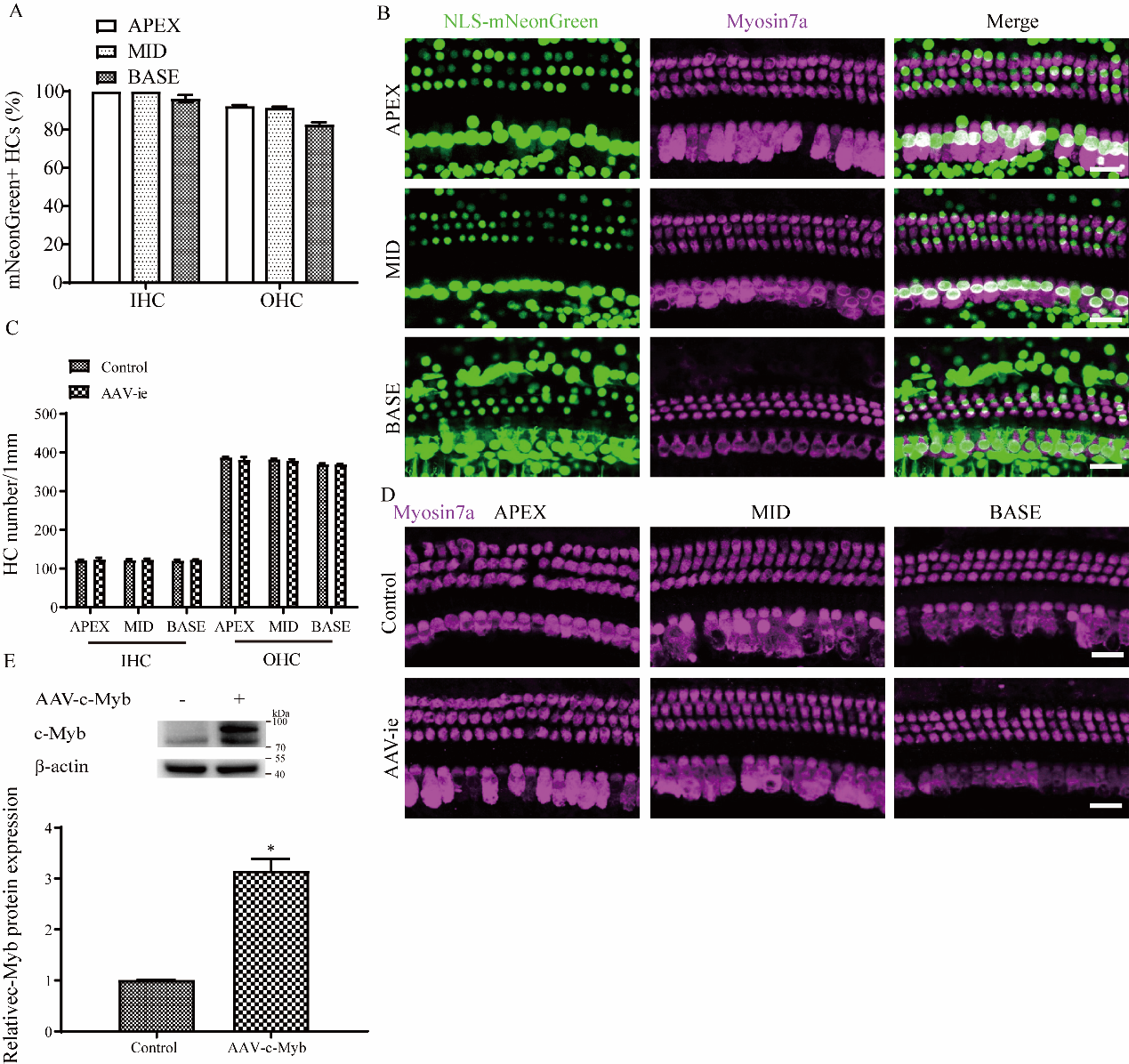


Supplementary Figure 2. **c-Myb expression is upregulated after AAV-c-Myb transfection in the cochlea through the RWM in mice.** To verify the efficiency of the viral transfection *in vivo*, mice were injected with 1×10^10^ GCs of AAV-ie into the left scala tympani through the left ear RWM at P16, and the mice were sacrificed at P30. (**A, B**) Immunofluorescence images showing the NLS-mNeon Green fluorescence (green) and myosin 7a staining (purple) in the apical, middle, and basal turns of cochleae infected with AAV-ie. The efficiencies of HC transfection in the apical, middle, and basal turns were 94.37±0.85%, 92.40±1.30%, and 84.3±2.60%, respectively. Scale bar = 20 μm. (**C, D**) Immunostaining and cell counting verified that there was no significant difference in HC number between the AAV-ie group and the control group. Scale bar = 20 μm. (**E**) The expression of c-Myb in cochlear HCs after the injection of 1×10^10^ GCs of AAV-c-Myb into the left scala tympani of mice at P16. Western blot showed that the protein level of c-Myb in cochlear HCs in the AAV-c-Myb group was significantly greater than that in the control group. ***p < 0.001, n = 3 for each group.


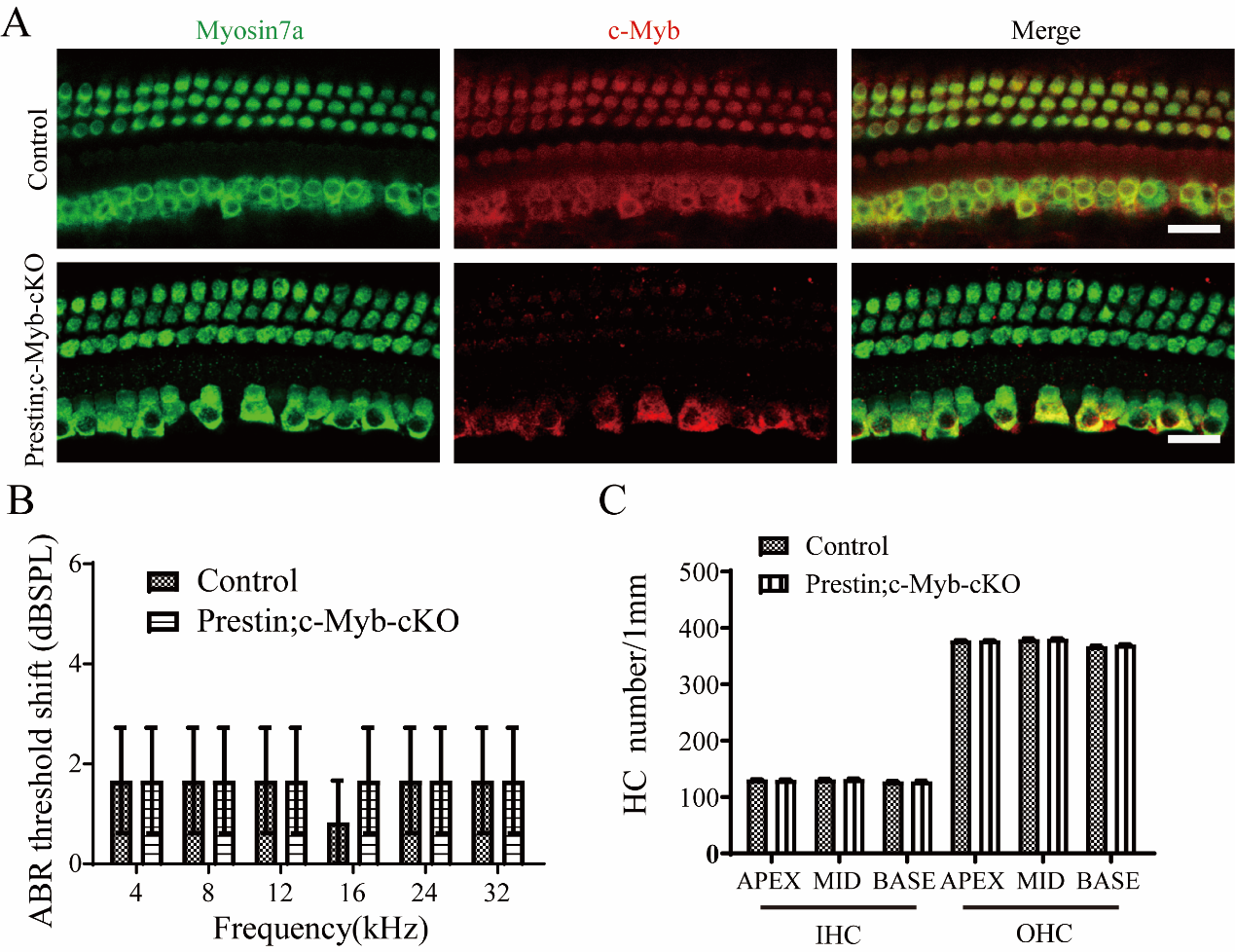


Supplementary Figure 3. **c-Myb-HC conditional knockout mice (Prestin; c-Myb-cKO) were generated by crossing c-Myb flox/flox mice with Prestin-CreER mice**. c-Myb-HC conditional knockout mice (Prestin; c-Myb-cKO) in which c-Myb expression is down-regulated only in cochlear OHCs were generated by crossing c-Myb flox/flox mice with Prestin-CreER mice. Tamoxifen was injected in mice at P10-P12 and the cochlea were harvest at P30. **(A)** Immunostaining showed that the c-Myb expression was significantly down-regulated in OHCs but not in IHCs in Prestin; c-Myb-cKO mice. **(B)** Hearing test showed that no statistical ABR threshold shift were found in the Prestin; c-Myb-cKO mice. **(C)** Cell counting showed that no statistical differences of HC numbers were found between the Prestin; c-Myb-cKO mice and control mice. Scale bar = 20µm.


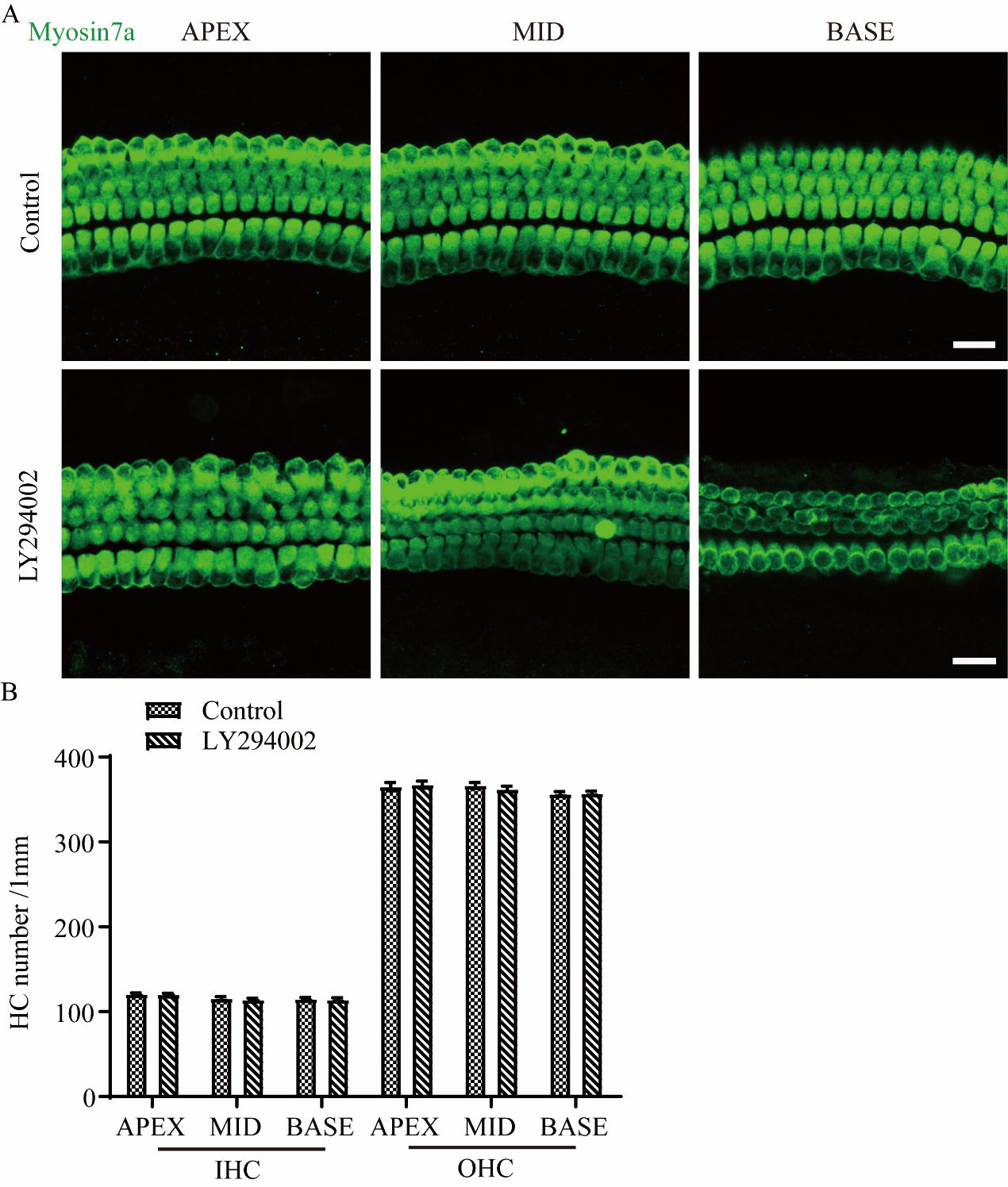


Supplementary Figure 4. **LY294002 alone treatment does not affect the viability of cochlear HCs.** The cultured HCs were treated with LY294002 (10 μM) for 50 h. **(A, B)** Immunofluorescence staining and cell counting showed that both the number of IHC and OHC were not changed significantly after LY294002 alone treatment compared with the control group. Scale bar = 20µm.
