# Supplementary material for: c-Myb protects cochlear hair cells from cisplatin-induced damage via the PI3K/Akt signaling pathway
Source: Cell Death Discov. 2022 Feb 24;8:78. doi: 10.1038/s41420-022-00879-9 (PMC8873213; doi:10.1038/s41420-022-00879-9)
Supplement: Supplementary file 2 — Supplementary tables [file 41420_2022_879_MOESM2_ESM.docx]

Table 1. The percentage of HC loss after cisplatin treatment *in vivo*

| % Distance from APEX | | Control(%) | Cisplatin(%) | Control (AAV-ie) (%) | Cisplatin + AAV-ie(%) | cisplatin + AAV-c-Myb(%) |
| --- | --- | --- | --- | --- | --- | --- |
| 6.66 | IHC | 0.000±0.000 | 0.980±0.620 | 0.000±0.000 | 0.490±0.490 | 0.000±0.000 |
|  | OHC | 0.290±0.183 | 0.290±0.183 | 0.290±0.183 | 0.290±0.183 | 0.290±0.183 |
| 13.32 | IHC | 0.490±0.490 | 0.490±0.490 | 0.490±0.490 | 0.490±0.490 | 0.490±0.490 |
|  | OHC | 0.435±0.195 | 0.435±0.195 | 0.290±0.183 | 0.580±0.290 | 0.290±0.183 |
| 19.98 | IHC | 0.490±0.490 | 0.490±0.490 | 0.490±0.490 | 0.490±0.490 | 0.490±0.490 |
|  | OHC | 0.290±0.183 | 0.580±0.290 | 0.290±0.183 | 0.580±0.290 | 0.290±0.183 |
| 26.64 | IHC | 0.490±0.490 | 0.490±0.490 | 0.490±0.490 | 0.490±0.490 | 0.490±0.490 |
|  | OHC | 0.435±0.195 | 0.725±0.145 | 0.580±0.183 | 0.725±0.349 | 0.725±0.145 |
| 33.30 | IHC | 0.490±0.490 | 0.980±0.620 | 0.490±0.490 | 0.980±0.620 | 0.490±0.490 |
|  | OHC | 0.580±0.183 | 0.870±0.318 | 0.580±0.183 | 0.870±0.225 | 0.580±0.183 |
| 39.96 | IHC | 0.980±0.620 | 0.980±0.620 | 0.490±0.490 | 0.980±0.620 | 0.490±0.490 |
|  | OHC | 0.580±0.183 | 1.015±0.267 | 0.580±0.183 | 1.015±0.145 | 1.015±0.267 |
| 46.62 | IHC | 0.980±0.620 | 1.470±0.657 | 0.490±0.490 | 0.980±0.620 | 0.490±0.490 |
|  | OHC | 0.580±0.183 | 1.015±0.145 | 0.870±0.000 | 1.015±0.145 | 1.160±0.183 |
| 53.28 | IHC | 0.980±0.620 | 0.980±0.620 | 0.980±0.620 | 0.980±0.620 | 0.980±0.620 |
|  | OHC | 0.580±0.183 | 0.870±0.000 | 0.725±0.145 | 0.870±0.318 | 1.015±0.349 |
| 59.94 | IHC | 0.980±0.980 | 1.470±0.657 | 0.980±0.620 | 0.980±0.620 | 0.980±0.620 |
|  | OHC | 0.870±0.318 | 1.305±0.195 | 0.725±0.145 | 1.305±0.373 | 1.160±0.367 |
| 66.60 | IHC | 0.980±0.980 | 1.470±0.657 | 0.980±0.620 | 1.960±1.518 | 0.980±0.620 |
|  | OHC | 0.870±0.318 | 1.305±0.195 | 1.015±0.349 | 1.160±0.290 | 1.450±0.183 |
| 73.26 | IHC | 0.980±0.620 | 2.940±0.000 | 0.980±0.620 | 3.920±0.980 | 1.470±0.657 |
|  | OHC | 1.885±0.267 | 5.800±0.535 | 1.740±0.318 | 5.655±0.435 | 1.595±0.415 |
| 79.92 | IHC | 1.960±0.980 | 5.880±1.073 | 1.960±1.518 | 6.860±1.518 | 1.960±1.518 |
|  | OHC | 2.175±0.195 | 12.171±0.978 | 2.320±0.485 | 12.171±1.365 | 3.335±0.415 |
| 86.58 | IHC | 2.450±1.180 | 11.275±1.597 | 2.940±1.074 | 11.763±1.075 | 5.390±1.073 |
|  | OHC | 3.190±0.485 | 23.768±2.319 | 3.045±0.490 | 24.783±2.139 | 4.495±1.015 |
| 93.24 | IHC | 3.920±0.980 | 22.550±1.454 | 3.920±1.518 | 21.570±3.561 | 5.390±2.213 |
|  | OHC | 3.335±0.349 | 39.855±2.136 | 3.480±0.502 | 41.595±2.526 | 6.670±0.996 |
| 99.90 | IHC | 4.410±1.004 | 39.218±1.808 | 3.920±0.620 | 39.708±4.462 | 7.350±1.259 |
|  | OHC | 4.060±0.580 | 60.435±3.009 | 3.915±0.490 | 59.855±2.762 | 8.407±1.466 |

Table 2. The percentage of HC loss in cochlea after cisplatin treatment *in vitro*

| % Distance from APEX | | Control(AAV-ie)(%) | Cisplatin+AAV-ie(%) | Cisplatin+AAV-c-Myb (%) |
| --- | --- | --- | --- | --- |
| 6.66 | IHC | 0.505±0.505 | 12.883±0.519 | 4.546±1.030 |
|  | OHC | 0.451±0.201 | 59.309±2.983 | 23.273±1.568 |
| 13.32 | IHC | 0.505±0.505 | 15.657±0.931 | 6.061±1.565 |
|  | OHC | 0.601±0.190 | 61.862±3.668 | 23.574±1.852 |
| 19.98 | IHC | 1.010±0.639 | 18.182±1.107 | 7.576±1.035 |
|  | OHC | 0.751±0.277 | 62.613±2.856 | 25.075±1.924 |
| 26.64 | IHC | 1.010±0.639 | 21.212±1.750 | 7.576±1.035 |
|  | OHC | 0.901±0.233 | 63.213±3.090 | 25.375±2.366 |
| 33.30 | IHC | 2.020±0.639 | 18.687±1.644 | 7.576±1.298 |
|  | OHC | 1.051±0.150 | 63.964±2.782 | 26.426±1.955 |
| 39.96 | IHC | 2.083±0.659 | 21.354±2.604 | 10.417±1.743 |
|  | OHC | 1.223±0.193 | 66.361±2.341 | 27.829±2.088 |
| 46.62 | IHC | 2.604±0.960 | 22.396±1.491 | 13.542±1.041 |
|  | OHC | 1.529±0.306 | 66.973±2.648 | 27.982±2.470 |
| 53.28 | IHC | 3.646±0.521 | 22.396±2.341 | 18.750±2.135 |
|  | OHC | 2.141±0.306 | 67.125±1.579 | 28.135±2.268 |
| 59.94 | IHC | 3.646±0.521 | 27.604±2.341 | 19.271±1.878 |
|  | OHC | 2.294±0.205 | 68.043±2.326 | 28.440±2.209 |
| 66.60 | IHC | 4.167±0.659 | 28.125±2.341 | 23.438±1.759 |
|  | OHC | 2.294±0.313 | 67.890±2.872 | 27.217±1.991 |
| 73.26 | IHC | 4.839±0.721 | 32.796±1.938 | 23.118±1.938 |
|  | OHC | 3.894±0.156 | 69.470±2.4922 | 29.907±1.619 |
| 79.92 | IHC | 4.301±0.680 | 32.796±1.938 | 24.194±1.997 |
|  | OHC | 4.050±0.197 | 69.938±2.745 | 29.907±2.289 |
| 86.58 | IHC | 4.839±0.721 | 36.022±3.051 | 26.344±1.938 |
|  | OHC | 4.050±0.394 | 71.184±2.191 | 30.685±2.282 |
| 93.24 | IHC | 4.839±0.721 | 39.247±3.051 | 26.344±2.416 |
|  | OHC | 5.140±0.319 | 70.094±2.554 | 30.997±2.547 |
| 99.90 | IHC | 5.376±1.075 | 42.473±2.688 | 26.882±2.452 |
|  | OHC | 0.075±0.526 | 70.249±1.796 | 32.555±2.191 |

Table 3. TUNEL-positive HC number, Cleavage-caspase3-positive HC number and Mito-SOX–positivie HC numbers in the cochlear basal turns and Ctbp2 puncta numbers per IHC.

| Positivie HC number and Ctbp2 puncta number | | Control (AAV-ie) | cisplatin + AAV-ie | Cisplatin + AAV-c-Myb |
| --- | --- | --- | --- | --- |
| TUNEL in vitro (HC/1 mm) | IHC | 0.293±0.186 | 4.908±0.378 | 1.913±0.267 |
|  | OHC | 1.772±0.326 | 10.410±0.814 | 6.642±0.514 |
| Cleaved Caspase3 in vitro(HC/1 mm) | IHC | 0.295±0.187 | 5.025±0.433 | 2.205±0.201 |
|  | OHC | 1.763±0.321 | 10.822±0.494 | 7.703±0.377 |
| Mito-SOX in vitro(HC/1 mm) | IHC | 0.293±0.186 | 5.610±0.372 | 2.347±0.294 |
|  | OHC | 1.903±0.274 | 11.392±0.434 | 4.993±0.434 |
| TUNEL in vivo(HC/1 mm) | IHC | 0.298±0.188 | 4.609±0.417 | 2.109±0.301 |
|  | OHC | 0.597±0.300 | 10.156±0.514 | 4.528±0.399 |
| Ctbp-2 in vivo (Ctbp2 puncta per IHC) | APEX | 15.667±0.333 | 8.500±0.224 | 13.333±0.333 |
|  | MID | 15.500±0.224 | 6.667±0.333 | 13.333±0.211 |
|  | BASE | 15.333±0.211 | 4.333±0.333 | 8.500±0.224 |

Table 4. The percentage of HC loss in cochlea *in vitro*

| % Distance from APEX | | Control(AAV-ie)( %) | Cisplatin+AAV-ie(%) | Cisplatin+AAV-c-Myb (%) | Cisplatin + AAV-c-Myb+LY294002(%) |
| --- | --- | --- | --- | --- | --- |
| 6.66 | IHC | 0.505±0.505 | 12.626±0.931 | 4.546±1.035 | 9.091±0.782 |
|  | OHC | 0.451±0.201 | 59.910±2.740 | 22.072±1.539 | 48.949±1.928 |
| 13.32 | IHC | 1.010±0.639 | 17.172±0.639 | 6.061±1.549 | 9.596±0.931 |
|  | OHC | 0.751±0.150 | 62.466±3.400 | 22.673±1.442 | 50.751±3.320 |
| 19.98 | IHC | 1.515±0.678 | 19.192±1.010 | 7.576±1.035 | 13.636±1.298 |
|  | OHC | 0.751±0.150 | 63.063±2.601 | 24.024±1.329 | 49.399±2.881 |
| 26.64 | IHC | 1.010±0.639 | 21.717±1.644 | 7.576±1.035 | 15.657±1.216 |
|  | OHC | 1.051±0.150 | 63.814±3.072 | 26.426±2.063 | 50.150±2.836 |
| 33.30 | IHC | 1.515±0.678 | 19.192±1.010 | 7.576±0.678 | 14.647±0.931 |
|  | OHC | 0.901±0.233 | 63.964±2.782 | 16.426±1.782 | 50.601±3.382 |
| 39.96 | IHC | 1.052±0.656 | 22.917±1.921 | 9.896±1.254 | 16.146±1.491 |
|  | OHC | 1.223±0.193 | 65.291±2.176 | 27.064±1.846 | 53.823±3.210 |
| 46.62 | IHC | 1.052±0.656 | 21.875±1.804 | 13.542±1.042 | 18.229±1.491 |
|  | OHC | 1.529±0.193 | 66.055±2.507 | 26.606±2.051 | 54.128±2.968 |
| 53.28 | IHC | 2.094±1.038 | 23.436±2.246 | 18.750±2.135 | 20.833±1.743 |
|  | OHC | 2.141±0.387 | 66.514±1.270 | 29.052±1.860 | 55.199±3.681 |
| 59.94 | IHC | 2.620±0.952 | 28.125±2.421 | 19.271±1.878 | 21.354±1.491 |
|  | OHC | 2.447±0.193 | 66.820±1.810 | 29.664±2.033 | 54.740±1.767 |
| 66.60 | IHC | 2.620±0.952 | 27.083±2.234 | 23.438±1.759 | 24.479±2.192 |
|  | OHC | 2.447±0.193 | 67.125±2.265 | 28.135±1.875 | 50.765±2.888 |
| 73.26 | IHC | 4.839±0.721 | 32.796±1.938 | 22.043±1.540 | 27.419±1.381 |
|  | OHC | 3.738±0.241 | 69.568±2.437 | 30.275±1.788 | 56.075±3.438 |
| 79.92 | IHC | 4.301±0.680 | 32.798±1.938 | 23.118±1.938 | 25.807±1.666 |
|  | OHC | 4.206±0.209 | 69.859±2.345 | 29.358±2.209 | 55.919±2.989 |
| 86.58 | IHC | 4.839±0.721 | 36.022±3.051 | 25.269±1.938 | 29.032±1.178 |
|  | OHC | 4.206±0.319 | 72.031±1.911 | 30.887±2.305 | 56.854±2.307 |
| 93.24 | IHC | 4.839±0.721 | 39.247±3.051 | 26.344±3.051 | 30.108±2.306 |
|  | OHC | 5.140±0.319 | 70.990±2.701 | 31.957±2.599 | 57.165±2.615 |
| 99.90 | IHC | 5.914±1.295 | 42.473±2.688 | 26.882±1.983 | 31.720±1.938 |
|  | OHC | 5.919±0.462 | 70.958±1.709 | 32.875±2.163 | 57.788±2.204 |
