# Supplementary material for: c-Myb protects cochlear hair cells from cisplatin-induced damage via the PI3K/Akt signaling pathway
Source: Cell Death Discov. 2022 Feb 24;8:78. doi: 10.1038/s41420-022-00879-9 (PMC8873213; doi:10.1038/s41420-022-00879-9)

**Uncropped western blots**

Figure 1D

The original image of the covered gel edge of Figure 1D are as follows：


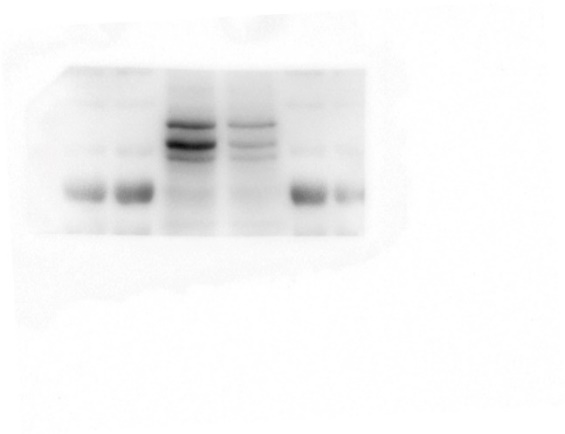

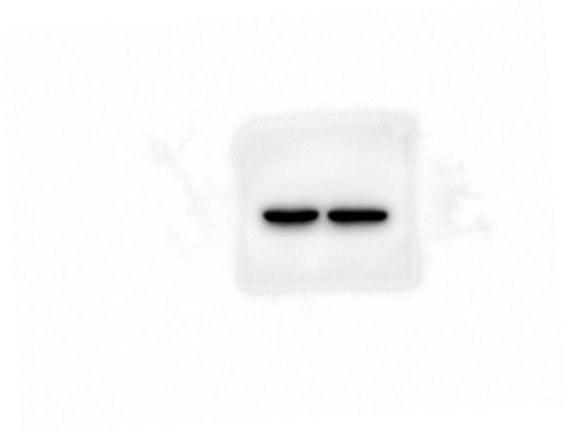


The original image of the covered gel edge of Figure 8A are as follows：


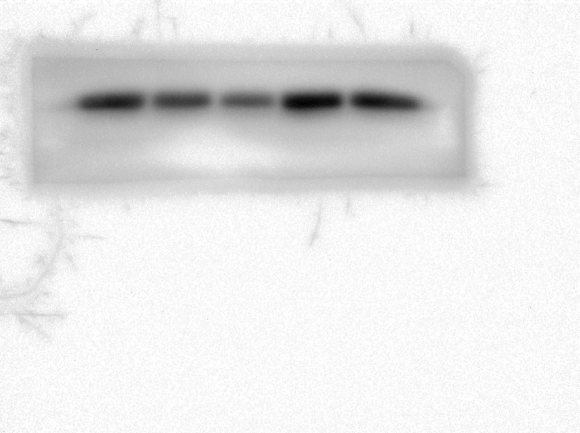

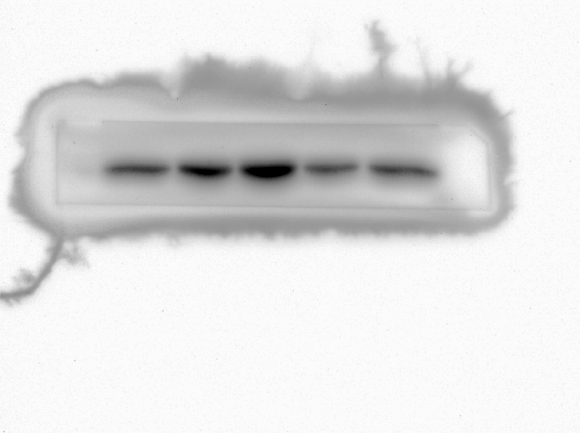


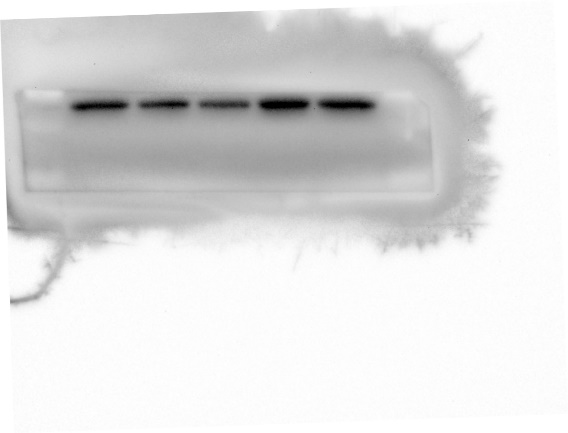

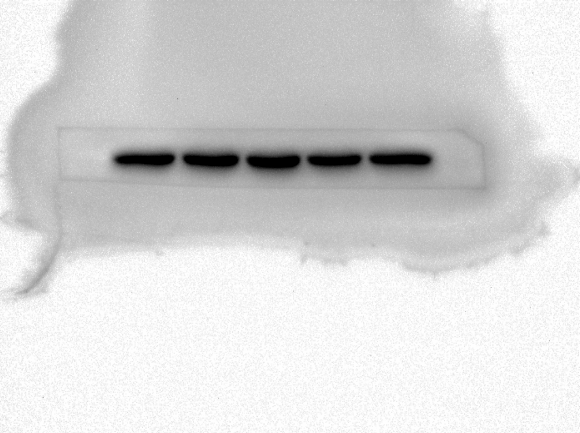


The original image of the covered gel edge of Supplementary Figure 1E are as follows：


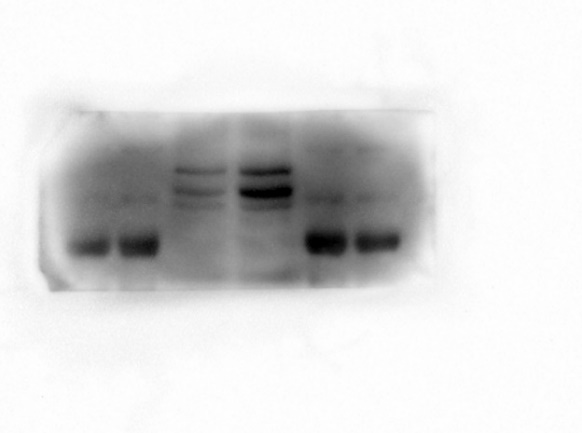

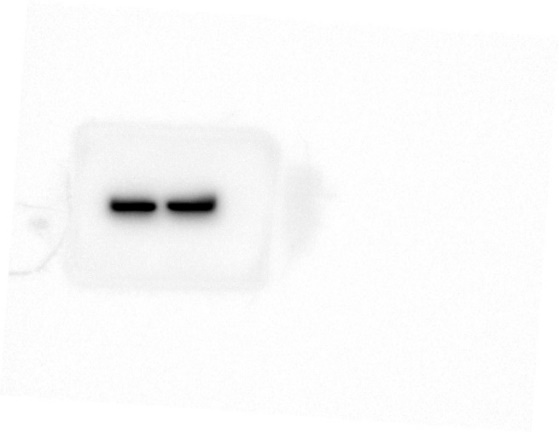


The original image of the covered gel edge of Supplementary Figure 2E are as follows：


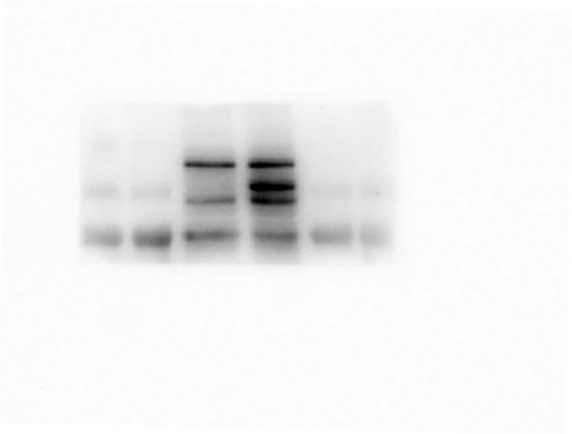

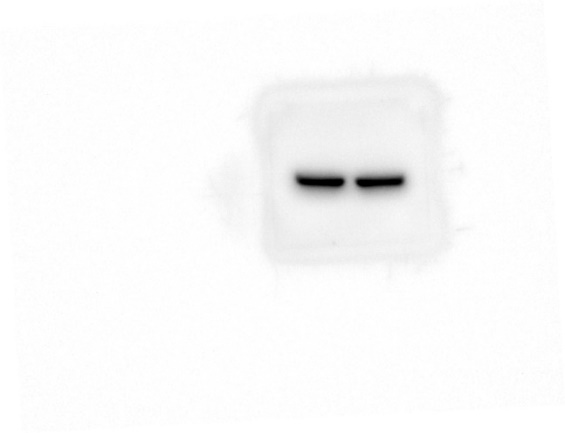

Supplement: Supplementary file 3 — uncropped western blots [file 41420_2022_879_MOESM3_ESM.docx]
